# Oxidation of the Platinum(II) Anticancer Agent [Pt{(*p*-BrC_6_F_4_)NCH_2_CH_2_NEt_2_}Cl(py)] to Platinum(IV) Complexes by Hydrogen Peroxide

**DOI:** 10.3390/molecules28176402

**Published:** 2023-09-01

**Authors:** Ruchika Ojha, Peter C. Junk, Alan M. Bond, Glen B. Deacon

**Affiliations:** 1 School of Chemistry, Monash University, Clayton, VIC 3800, Australia; ruchika.ojha@monash.edu (R.O.); alan.bond@monash.edu (A.M.B.); glen.deacon@monash.edu (G.B.D.); 2 College of Science, Technology & Engineering, James Cook University, Townsville, QLD 4811, Australia

**Keywords:** platinum anticancer agents, platinum(II) complexes, platinum(IV) complexes, hydrogen peroxide oxidation of platinum(II) complexes

## Abstract

Pt^IV^ coordination complexes are of interest as prodrugs of Pt^II^ anticancer agents, as they can avoid deactivation pathways owing to their inert nature. Here, we report the oxidation of the antitumor agent [Pt^II^(*p*-BrC_6_F_4_)NCH_2_CH_2_NEt_2_}Cl(py)], **1** (py = pyridine) to dihydroxidoplatinum(IV) solvate complexes [Pt^IV^{(*p*-BrC_6_F_4_)NCH_2_CH_2_NEt_2_}Cl(OH)_2_(py)].H_2_O, **2·H_2_O** with hydrogen peroxide (H_2_O_2_) at room temperature. To optimize the yield, **1** was oxidized in the presence of added lithium chloride with H_2_O_2_ in a 1:2 ratio of Pt: H_2_O_2,_ in CH_2_Cl_2_ producing complex **2·H_2_O** in higher yields in both gold and red forms. Despite the color difference, red and yellow **2·H_2_O** have the same structure as determined by single-crystal and X-ray powder diffraction, namely, an octahedral ligand array with a chelating organoamide, pyridine and chloride ligands in the equatorial plane, and axial hydroxido ligands. When tetrabutylammonium chloride was used as a chloride source, in CH_2_Cl_2_, another solvate, [Pt^IV^{(*p*-BrC_6_F_4_)NCH_2_CH_2_NEt_2_}Cl(OH)_2_(py)].0.5CH_2_Cl_2,_ **3·0.5CH_2_Cl_2_**, was obtained. These Pt^IV^ compounds show reductive dehydration into Pt^II^ [Pt{(*p*-BrC_6_F_4_)NCH=CHNEt_2_}Cl(py)], **1H** over time in the solid state, as determined by X-ray powder diffraction, and in solution, as determined by ^1^H and ^19^F NMR spectroscopy and mass spectrometry. **1H** contains an oxidized coordinating ligand and was previously obtained by oxidation of **1** under more vigorous conditions. Experimental data suggest that oxidation of the ligand is favored in the presence of excess H_2_O_2_ and elevated temperatures. In contrast, a smaller amount (1Pt:2H_2_O_2_) of H_2_O_2_ at room temperature favors the oxidation of the metal and yields platinum(IV) complexes.

## 1. Introduction

The serendipitous discovery of cisplatin as an anticancer drug has served humankind for decades [1,2]. Despite its success in treating testicular, ovarian, head and neck, small-cell lung, and bladder cancers, with a 90% cure rate for testicular cancer [3,4,5,6,7,8], cisplatin still has limited applications owing to natural and acquired resistance against tumors [9,10]. Additionally, it causes severe side effects, such as nephrotoxicity, neurotoxicity, and myelosuppression [9,10,11,12,13,14,15,16], and it can only be administered intravenously. The need to address these limitations and increase their applications to a broader spectrum of cancers produced cisplatin derivatives and new Pt^II^ compounds. However, only Pt^IV^ compounds showed the potency to shift the paradigm because their relatively inert nature reduces the extent of side reactions—it makes them less toxic and provides the possibility of their oral administration [8,17,18,19,20] for improved quality of life of cancer patients. Furthermore, they are more water-soluble than Pt^II^ drugs and are more easily absorbed in the gastrointestinal tract; their high lipophilicity causes enhanced diffusion through the cell membrane, and they are not cross-resistant with cisplatin [21,22,23].

Generally, Pt^IV^ compounds (e.g., tetraplatin, iproplatin, and satraplatin) are considered to be Pt^II^ prodrugs [24,25]. They are relatively inert to ligand substitution reactions due to their electronic configuration, but liable to two-electron reduction. They are reduced to their active Pt^II^ analogue in vivo by biomolecules, such as glutathione (GSH), by the loss of their axial ligands [8,26,27]. The ease with which Pt^IV^ complexes are reduced to Pt^II^ depends on the nature of the axial ligands [28], which affect the reduction potential (*E^red^*) for the Pt^IV/II^ process. For example, the order of the ease of reduction of Pt^IV^ [28] in tetraplatin (axial ligand Cl^−^) [29], JM216 (axial ligand CH_3_COO^−^) [30], and Iproplatin (axial ligand OH^−^) [31] is Cl^−^ > RCO_2_^−^ > OH^−^. Photoactive Pt^IV^ complexes have also been explored for better efficiency [32,33]. Nanoparticle-based drug delivery of Pt^IV^ complexes has been introduced to address the resistance issues and further lower the toxicity [34,35]. In most cases, the reduction of the Pt^IV^ prodrug generates the parent Pt^II^ compound by the loss of two axial ligands [27,36]. Some reports also mentioned the formation of more than one reduction product, depending on the reducing agents used [37,38].

**Figure 1:**
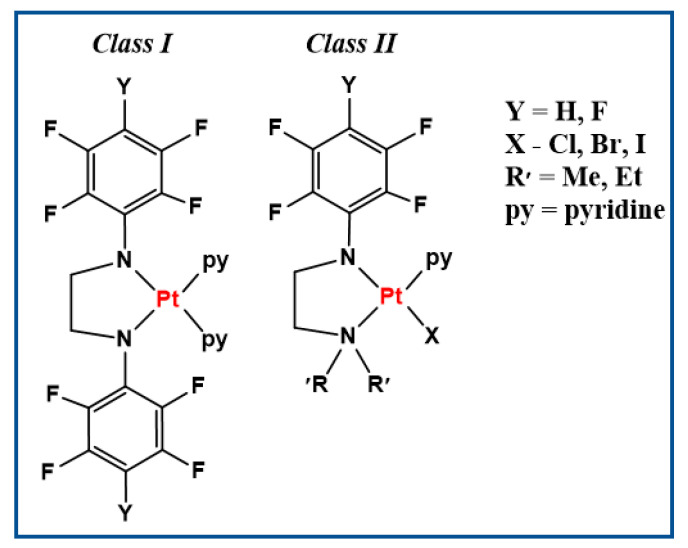
*Class 1* and *Class 2* organoamidoplatinum(II) compounds.

The need to find better drugs led to “rule breaker” [39] or “non-traditional” drugs [40], which violate structure–activity rules [41]. One such example is the polynuclear platinum compound BBR3464 [42,43,44]. Other “rule breakers” include two classes of organoamidoplatinum(II) compounds, namely, *Class 1*, [Pt{N(R)CH_2_}_2_(py)_2_] (R = polyfluoroaryl), with no H atoms on the N-donor atoms, and *Class 2*, *trans*-[Pt{N(R) CH_2_CH_2_NRʹ_2_}X (py)] (R = polyfluoroaryl; R′ = Et, Me; and X = Cl, Br, I), with *trans* amine ligands and *trans* anionic ligands, and no H atoms on the N-donor atoms (see Figure 1). Both classes show promising anticancer activity in vitro and in vivo [45,46,47]. The investigation of DNA binding properties by Resonant X-ray emission spectroscopy (RXES) shows that after hydrolysis of [Pt{N(*p*-HC_6_F_4_)CH_2_}_2_(py)_2_] (Pt-103) (py = pyridine), the [N(*p*-HC_6_F_4_)CH_2_]_2_^2−^ moiety behaves as a leaving group, and hydroxylation of the Pt center generates a hydrolyzed species, which then reacts with DNA and preferentially coordinates to the adenine site, rather than the guanine site [48]. This could explain why Pt-103 is biologically active against cisplatin-resistant cell lines.

Pt^IV^ compounds are generally six coordinate octahedral complexes and can be synthesized by oxidative addition of four coordinate square planar Pt^II^ compounds. However, some five coordinat Pt^IV^ complexes also have been reported to be formed in the oxidation of Pt^II^ to Pt^IV^ [49]. The Platinum Group Metals (PGMs) in higher oxidation states (as Pt^IV^) have been reported as possible intermediates in some organic synthetic procedures due to their lower stability [50,51,52,53].

**Scheme 1:**
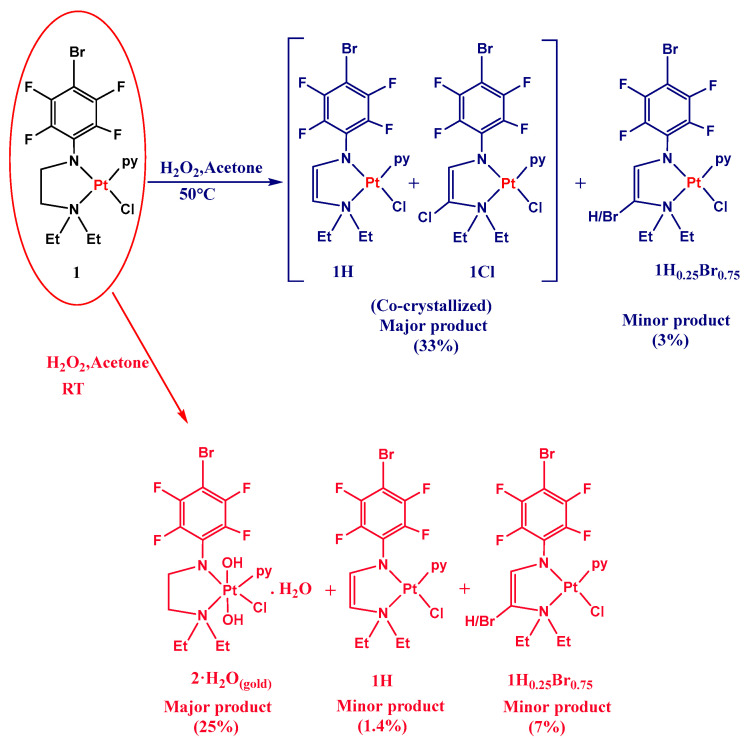
A comparison of the products obtained from the H_2_O_2_ oxidation of **1** in acetone, with heating (top blue) and at room-temperature (bottom red) conditions.

Oxidation of platinum(II) complexes with H_2_O_2_ has been reported to produce *trans* dihydroxidoplatinum(IV) complexes [54,55,56,57,58,59,60]. Some *Class I* (organoamido)platinum(IV) compounds have shown high anticancer activity in vivo against the ADJ/PC6 tumor line [54]. Some Pt^IV^ complexes with large negative *E^red^* values, such as [Pt{((*p*-HC_6_F_4_)NCH_2_)_2_(py)_2_}(Cl)_2_], [Pt{((*p*-HC_6_F_4_)NCH_2_)_2_(py)_2_}(OH)_2_] (Pt103(OH)_2_), and [Pt{((*p*-HC_6_F_4_)NCH_2_)_2_(py)_2_}(Cl)(OH)], are effective against cisplatin-resistant cell lines [54]. The last two are even more active than the Pt^II^ precursor Pt-103. This suggests that either the Pt^IV^ complexes themselves are active, or they are reduced in vivo near the target and prevent premature reduction [54]. The electrochemical oxidation of *Class 2* complexes produced mononuclear, formally Pt^III^, species [61,62]. The formation of Pt^IV^ species was not observed in these electrochemical studies. Recently, we reported the oxidation of the *Class 2* complex [Pt(*p*-BrC_6_F_4_)NCH_2_CH_2_NEt_2_}Cl(py)], (**1**) by an excess of H_2_O_2_ under forcing conditions, which involved heating in different solvents [47]. Under these conditions, two types of ligand oxidized organoenamineamidoplatinum(II) complexes, namely, unsubstituted [Pt(*p*-BrC_6_F_4_)NCH=CHNEt_2_}Cl(py)], **1H** and ethene-substituted [Pt(*p*-BrC_6_F_4_)NCH=C(X)NEt_2_}Cl(py)], **1X** (X = Cl, Br), along with [Pt(*p*-BrC_6_F_4_)NCH=C(H_0_._25_Br_0.75_)NEt_2_}Cl(py)], **1H_0.25_1Br_0.75_**, were obtained. (Scheme 1 top) [47]. However, the formation of Pt^IV^ complexes was not observed.

Here, we report the formation of Pt^IV^ dihydoxidoplatinum(IV) complexes, which are isolated from the oxidation of **1** with H_2_O_2_ under milder conditions without heating. Changes in stoichiometry, temperature, solvents, and the addition of chloride were used to achieve higher selectivity of the products. These Pt^IV^ compounds undergo reductive dehydration, that is, reduction of Pt^IV^ to Pt^II^ over time in the solid state, as determined by X-ray powder diffraction characterization, forming organoenamineamideplatinum(II) complexes with an oxidized coordinating ligand.

## 2. Results and Discussion

In the present work, the chemical oxidation of **1** was undertaken with H_2_O_2_ at room temperature in a range of solvents to determine if isolable Pt^IV^ complexes could be obtained under mild conditions. The H_2_O_2_ oxidation in acetone produced the gold-colored dihydroxidoplatinum(IV) compound **2·H_2_O_(gold)_** as a hydrate, along with **1H**, and **1H_0.25_1Br_0.75_** organoenamineamidoplatinum(II) compounds as minor products (Scheme 1(bottom, red)).

The Pt^IV^ complex **2·H_2_O_(gold)_** showed slow reductive dehydration in the solid state to produce the organoenamineamide Pt^II^ complex **1H**, which is the product of oxidation of the ligand [47] (see below). Earlier studies showed that some Pt^IV^ complexes also exhibit reductive elimination similar to what is better known for Pd^IV^ complexes [50].

**Scheme 2:**
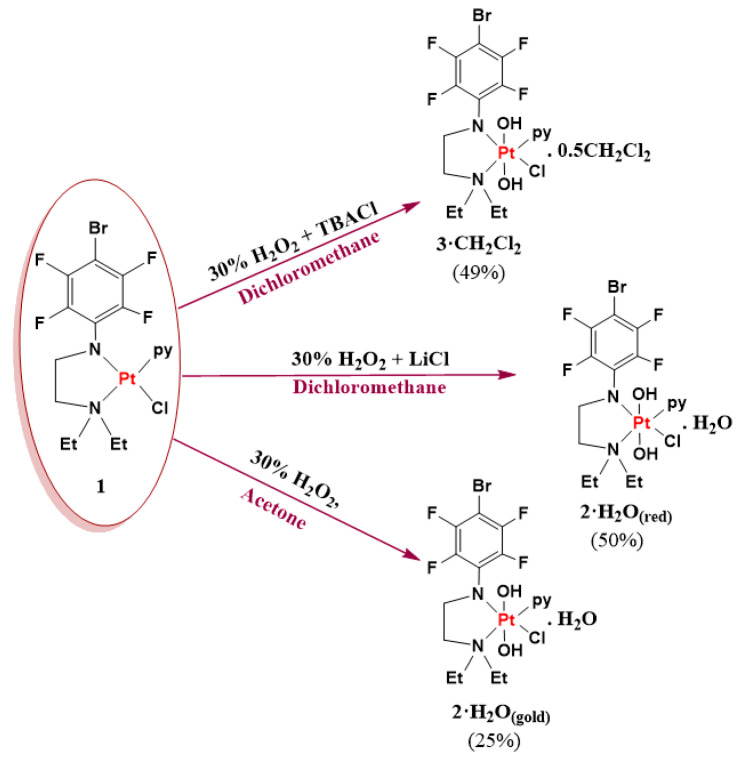
Oxidation of 1 with H2O2 under mild conditions showing major products.

The selectivity for formation of Pt^IV^ compounds was enhanced by optimizing the experimental conditions to produce the outcome shown in Scheme 2. We proposed previously that, in the absence of any other source of Cl^−^ in the reaction mixture, the chloride needed to form **1Cl** is generated from the oxidation of the Pt-Cl bond of **1** [47]. Thus, H_2_O_2_ oxidation reactions of **1** were also performed with deliberately added chloride to examine whether this would enhance the yield of **1Cl** or would promote the formation of Pt^IV^ complexes, as the Pt-Cl bond should remain intact due to the presence of excess chloride. Scheme 2 is a schematic representation of the major products obtained from the oxidation of **1** with limited H_2_O_2_ under a range of experimental conditions. Two differently colored samples of **2·H_2_O**, red and gold (**2·H_2_O_(red)_** and **2·H_2_O_(gold)_**), were isolated. The amounts of the reagents and the yields of the products are shown in Table 1. The results demonstrate that the addition of chloride enhances the formation of dihydroxidoplatinum(IV) complexes.

The H_2_O_2_ oxidation reaction of **1** in the presence of LiCl in CH_2_Cl_2_ at room temperature produced the Pt^IV^ complex **2·H_2_O** in two colors, red and golden crystals (see experimental section). Both were characterized by X-ray crystallography (see below) and microanalyses. Both have one molecule of water of crystallization per molecule of the complex (some previous works have also reported the observation of transient red color upon oxidation of cisplatin with chlorine [63,64]). Related results were obtained with added tetrabutylammonium chloride, in CH_2_Cl_2_ (see Table 1 and Experimental section). In this case, a deep-red-colored Pt^IV^ complex containing 0.5 of a CH_2_Cl_2_ molecule in the asymmetric unit, **3·0.5CH_2_Cl_2_**, with some **2·H_2_O_(gold)_** was obtained. A few crystals of NBu_4_[PtCl_3_(py)] also were obtained and characterized by X-ray crystallography only. All the experimental results suggest that adding chloride simplifies the reaction by preventing the dissociation oxidation of the Pt-Cl bond.

The mechanism for H_2_O_2_ oxidation of Pt^II^ to Pt^IV^ has been reported as single-step two-electron oxidation or as two very rapid one-electron processes that exclusively produce *trans*-hydroxide complexes [65]. In this oxidation reaction, the square planar configuration of the original Pt^II^ complex is retained, and the two hydroxido ligands coordinate *trans* to each other. A ^195^Pt NMR study has suggested that one of the *trans*-coordinated hydroxido ligands originates from H_2_O_2_ and the other from the solvent water [66,67].

Notably, all Pt^IV^ solvate complexes show reductive dehydration, i.e., from Pt^IV^ to Pt^II^ species. However, the resulting Pt^II^ species is not the parent compound **1**, as is generally the case with Pt^IV^ species. Instead, an organoenamineamidoplatinum(II) compound **1H** with an oxidized coordinating ligand is formed (below).

### 2.1. X-ray Crystal Structures

**Figure 2:**
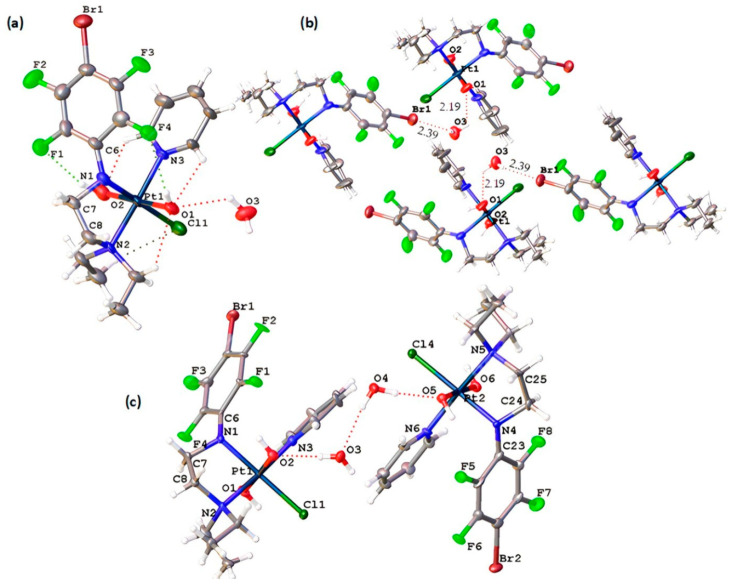
Molecular structures of Pt^IV^ complexes (**a**) **2·H_2_O_(gold)_** with intramolecular H-bonding; (**b**) crystal packing in **2·H_2_O_(gold)_** showing intermolecular H-bonding; and (**c**) **2·H_2_O_(red)_**, with 50% thermal ellipsoids.

**Figure 3:**
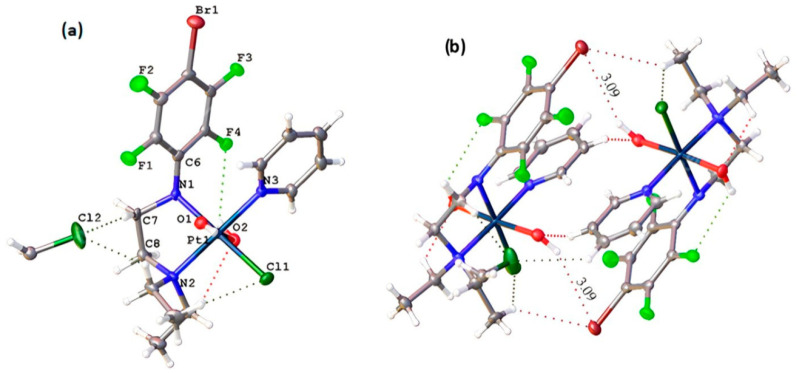
(**a**) Molecular structure of Pt^IV^ complex **3·0.5CH_2_Cl_2_**, showing 50% thermal ellipsoids. (**b**) Crystal packing in **3·0.5CH_2_Cl_2_**, showing inter- and intramolecular H-bonding.

The molecular structures of **2·H_2_O_(red)_** and **2·H_2_O_(gold)_** Pt^IV^ complexes are shown in Figure 2, and that of **3** in Figure 3. **2·H_2_O_(gold)_** crystallizes in the space group (*C*2/*c*), whereas **2·H_2_O_(red)_** crystallizes in the *Cc* space group. Despite these space group differences, their unit cells are virtually identical (Table 2), as are their X-ray powder diffractograms generated from their single-crystal data (Figure S1). Accordingly, the structures are the same, despite the optimum solutions being in different space groups. The molecular structure of **2·H_2_O_(red)_** has the same ratio of complex to solvent of crystallization (water) as in **2·H_2_O_(gold)_**. In **2·H_2_O_(red)_**, two dihydroxidoplatinum(IV) molecules bridged with two water molecules by H-bonding are present in the asymmetric unit (Figure 2c), whereas in **2·H_2_O_(gold)_**, a single molecule of **2** is associated with a single water molecule of crystallization (Figure 2a). However, the crystal structure of **2·H_2_O_(gold)_** extended to two asymmetric units (Figure 2b) reveals the same structure as in the asymmetric unit of **2·H_2_O_(red)_**. Red and yellow compounds with essentially the same structure are observed in the case of yellow and red mercuric oxide, ref [68] where the difference is attributed to particle size. Likewise, [RuBr_2_(CO)_2_(tpy)] (tpy = 2,2′:6′,2′′-terpyridine) exists in red and yellow forms with essentially the same structure [69]. Although the powder diffractograms of bulk **2·H_2_O_(red)_** and **2·H_2_O_(gold)_** show significant differences (Figure S7), this is attributed to different rates of reductive dehydration in the solid state (see below). **3·0.5CH_2_Cl_2_** crystallizes in the *C*2/*c* space group with one formula unit in the asymmetric unit (Figure 3a), and the crystal packing shows a hydrogen-bonded dimer (Figure 3b).

All Pt^IV^ solvate complexes have an octahedral stereochemistry around the Pt metal atom with *trans* hydroxido axial ligands. The bond lengths are listed in Table 3, the bond angles are in Tables S1 and S2, and the crystal data are in Table 2. The OPtO angles in **2·H_2_O_(gold)_**, **2·H_2_O_(red_**_)_, and **3·0.5CH_2_Cl_2_** are 177.7(3), 177.9 (3)/ 177.8(4) (molecules A and B), and 176.57(13), respectively. These angles are comparable to 179.73(9) in [Pt{((*p*-HC_6_F_4_)NCH_2_)_2_}(py)_2_(OH)_2_] ([Pt103(OH_2_)]) [54], as expected for a linear and *trans* arrangement of axial ligands. Equatorial positions have a square planar arrangement of the donor atoms, as in the parent compound **1**. The Pt-O bond lengths shown in Table 3 are similar to those for the dihydroxidoplatinum(IV) complex, [Pt103(OH_2_)] [54], shown in Table 3. The Pt-N bond lengths are expected to lengthen with an increase in the coordination number from 4 to 6. However, an increase in the oxidation state from +2 to +4 is expected to shorten the Pt-N bond lengths. Apparently, these contrary effects cancel each other out, so the Pt-N bond lengths remain close to those of the parent platinum(II) compound **(1)** at the three-esd level. The angles between OH and other donor atoms in the square planar arrangement are close to 90°, consistent with octahedral stereochemistry.

The bond angles around the amide N in **2·H_2_O_(red)_)** (108.8 (7) + 117.4 (8) + 118.2 (7) = 344.4°) and in **3** (108.054 (18) + 117.856 (17) + 116.878 (6) = 344.8°) show distortion from tetrahedral (∑328.5°) towards triangular (∑360°). However, the N atoms have less trigonal character than in the platinum(II) complexes **1** (∑356.9°) and **1H** (∑357°).

These Pt^IV^ complexes show a range of inter- and intramolecular H-bonding (Figure 2 and Figure 3), as also observed for related Pt^II^ compounds [70]. The interaction distances are listed in Table 4. In **2·H_2_O_(red)_** and **2·H_2_O_(gold),_** both H atoms of the *trans*-OH groups are facing toward the *o*-F atoms of the polyfluoroaryl ring. However, in **3**, one of the H atoms of the *trans*-OH ligands, faces away, as it makes an H-bond with Br(*p*-BrC_6_F_4_) of an adjacent molecule with an H⋯Br distance, 3.0993(5), as shown in Figure 3.

A few crystals of [NBu_4_][PtCl_3_(py)] were obtained as a minor product from the H_2_O_2_ oxidation reaction of **1** with added tetrabutylammonium chloride in CH_2_Cl_2_ (see Experimental). The compound [NBu_4_][PtCl_3_(py)] could only be characterized by X-ray crystallography due to the poor yield. The molecular structure of [NBu_4_][PtCl_3_(py)] is shown in Figure S2. In the asymmetric unit, only half of the molecule is present, and the other half is symmetrically generated. Crystallographic data are given in Table S3, and selected bond lengths and angles are listed in Table S4.

Satisfactory microanalyses were obtained for all three **2·H_2_O_(red)_**, **2·H_2_O_(gold)_**, and **3·0.5CH_2_Cl_2_** Pt^IV^ complexes. All were consistent with the presence of H_2_O in **2** and 0.5 CH_2_Cl_2_ in **3** crystal lattices (see Experimental section). The characteristic ν(OH) bands of dihydroxidoplatinum(IV) complexes were observed in IR spectra between (3500 and 3700 cm^−1^) in the expected range (ν(OH) 3760–3500 cm^−1^) for metal hydroxides [54,71]. Strong ν(C-F) absorption bands appear at a comparatively higher wavenumber, i.e., at 968 cm^−1^ in **2_(red)_** and **3** as compared to the Pt^II^ precursor **1** (956 cm^−1^), as also observed for Pt103 (926 cm^−1^) and Pt103(OH)_2_ (938 cm^−1^) [54]. The spectra show C-H out-of-plane deformation bands of py at 763 cm^−1^.

### 2.2. Powder X-ray Diffraction (PXRD) Study

Powder diffraction patterns for bulk **2·H_2_O_(red)_** and **2·H_2_O_(gold)_** exhibit differences (Figure S7) and also differ from the identical patterns generated from single-crystal data (Figure S1). These solids, especially red **2·H_2_O**, changed in physical appearance with time (over almost 90 days) and turned from deep-red crystals (block) into a mixture of red powder and some yellow solid.

A comparison of the PXRD pattern for a bulk sample of **2·H_2_O_(red)_** with those calculated from the single-crystal data for the platinum(II) complex [Pt(*p*-BrC_6_F_4_)NCH=CHNEt_2_}Cl(py)], **1H** [47] (Scheme 1) and single crystals of **2·H_2_O_(red)_** (see Figure S8) clearly shows that Pt^IV^ **2·H_2_O_(red)_** and **1H** both are present in the bulk sample of stored **2·H_2_O_(red)_**. A comparison between the PXRD patterns of the bulk sample of **2·H_2_O_(gold)_** with those calculated from the single-crystal data for **1H** and single crystals of **2·H_2_O_(gold),_** (Figure S9), clearly shows **2·H_2_O_(gold)_** and **1H** both are present in the bulk sample of **2·H_2_O_(gold)_**. However, **1H** is present in larger amounts in this bulk sample than in the bulk sample of **2·H_2_O_(red)_**.

All observations and the experimental facts confirm that these Pt^IV^ samples undergo reductive dehydration to produce a Pt^II^ complex with an oxidized ligand, where the rate is faster for **2·H_2_O_(gold)_** than for **2·H_2_O_(red)_**.

A yellow crystal of **1H** was collected from the bulk sample of **2·H_2_O_(red)_**, and X-ray diffraction data were obtained (see Table S5). The crystal structure showed twinning, but the cell parameters were consistent with **1H** [47]. Another yellow crystal of co-crystallized ([Pt(*p*-BrC_6_F_4_)NCH=CHNEt_2_}Cl(py)], **1H** and [Pt(*p*-BrC_6_F_4_)NCH=C(Cl)NEt_2_}Cl(py)], **1Cl**, **1H/Cl**) also was collected from a bulk sample of **2·H_2_O_(gold)_**, and X-ray diffraction data were obtained (see Table S5). The crystal structure showed twinning, but the cell parameters were consistent with co-crystallized **(1H/Cl)** [47]. The mechanism for formation of **1Cl** by reductive dehydration in the solid state is not fully understood, but it is probably via a similar path to that proposed for its formation in the oxidation of **1** by H_2_O_2_ under aggressive conditions [47].

### 2.3. Isolation of PtIV from the Solution of an Aged Bulk Sample

To examine if platinum(IV) species can be recovered from solution, an aged sample of **3·0.5CH_2_Cl_2_** was returned to the reaction mixture from which **3·0.5CH_2_Cl_2_** was isolated in the first place and was completely dissolved in CH_2_Cl_2_ to obtain an homogeneous solution. Crystallization from the CH_2_Cl_2_/hexane mixture enabled the isolation of red crystals of [Pt^IV^{(*p*-BrC_6_F_4_)NCH_2_CH_2_NEt_2_}Cl(OH)_2_(py)].H_2_O, **2·H_2_O_(red)_** having the same cell parameters (see Table S6) as in Table 2. However, **3** (with 0.5 CH_2_Cl_2_ in the crystal lattice) could not be isolated. A yellow crystal of co-crystallized **(1H/Cl)** again was isolated from the solution, and the unit cell parameters collected (Table S6) are in agreement with those reported in Table S5. Some pale-yellow crystals were also isolated from the solution, and X-ray diffraction data were obtained, establishing the identification as [NBu_4_][PtCl_3_(py)] (Figure S2), as also isolated from the preparation of **3·0.5CH_2_Cl_2_** (above).

### 2.4. NMR Spectroscopy

The rearrangement of the Pt^IV^ complexes into the Pt^II^ species, **1H**, was initially detected by NMR spectroscopy. Notably, no Pt^IV^ species was detected in the NMR spectra of **2·H_2_O_(gold)_**, and only **1H** was observed (see Experimental section), showing that the rate of reductive dehydration for **2·H_2_O_(gold)_** is fast in solution. In the ^19^F NMR spectrum of **2·H_2_O_(red)_** in deuterated acetone, four resonances in a 1:1:1:1 ratio are present at −138.24, −138.34, −140.64, and −148.16 ppm, together with very low-intensity resonances of precursor compound **1**. The highest and lowest frequency resonances correspond to those of **1H [47]**. The other two resonances show the small separation observed for F2,6 and F3,5 of the *p*-HC_6_F_4_ group in other 2,3,5,6-tetrafluorophenylethane-1,2-diaminatoplainum(IV) complexes [54]. The signals are shifted to a higher frequency than for the corresponding resonances of the Pt^II^ precursor due to the higher oxidation state.

In the ^1^H NMR spectrum of **2·H_2_O_(red)_** in (CD_3_)_2_CO, two separate resonance sets were observed for *ortho*, *meta,* and even for the *para* protons of the pyridine ligands. That is only possible when two species are present in the solution. One set of resonances corresponds to those reported for **1H** [47]. The remaining resonances are attributed to those of the platinum(IV) complex **2** in a 1:1 ratio with **1H**. Further to this assignment, in the ^1^H NMR spectra, one of the two sets of pyridine ^1^H resonances appears at a higher frequency than the other set. This set of resonances is assigned to pyridine of a platinum(IV) species. ^1^H and ^19^F NMR spectra of **2·H_2_O_(red)_** in CD_2_Cl_2_ also showed both species, confirming that this observation is not solvent-specific.

Similar behavior was shown by **3·0.5CH_2_Cl_2_**. In the solution, **3·0.5CH_2_Cl_2_** produces a Pt^IV^ species and **1H**, as observed for **2·H_2_O_(red)_**. However, this compound also shows the chloro-substituted organoenamineamide species [Pt(*p*-BrC_6_F_4_)NCH=C(Cl)NEt_2_}Cl(py)], **1Cl [47]** in solution. In the ^19^F NMR spectrum, the resonances at −137.96 and −148.61 ppm (almost 10 ppm apart) are due to F 3, 5 (2F) and F 2, 6 (2F) of **1H**. Those at −137.63 and −142.25 ppm (almost 4 ppm apart) represent 2F (F 3, 5) and 2F (F 2, 6) of the Pt^IV^ species, **3**, and at −137.27 and −148.46 ppm (almost 10 ppm apart), they are 2F (F 3, 5) and 2F (F 2, 6) of **1Cl**. Some extracted data from complex NMR spectra of these compounds are shown in Table 5. For **2·H_2_O_(gold)_**_,_ only **1H** was observed, and the Pt^IV^ complex was not observed; therefore, only the data for **1H** are shown in Table 5. The complete NMR data and integrations showing relative amounts are provided in the Experimental section.

All the above observations indicate that Pt^IV^ complexes show reductive dehydration to Pt^II^ species, mainly **1H,** in solution (Equation (1)) and in the solid state. In this rearrangement, the metal is reduced, and the ligand is oxidized with water loss. This is a net dehydration reaction, as given in Equation (1). The rate at which Equation (1) occurs is strongly dependent on the environment. After the peroxide reaction, these Pt^IV^ complexes were isolated with the addition of water (see Experimental section), so Equation (1) is not favored. In the solid state, slow loss of water or dehydration occurs. In an organic solvent with minimal water content, water loss is facilitated and faster than in the solid state.

(1) $$\left[{Pt}^{IV}\left\{\left(p-{BrC}_{6}F_{4}\right){NCH}_{2}{CH}_{2}{NEt}_{2}\right\}Cl\left(OH{)}_{2}\left(py\right)\right]⟶{Pt}^{II}\left\{\left(p-{BrC}_{6}F_{4}\right)NCH={CHNEt}_{2}\right\}Cl\left(py\right)\right]+2H_{2}O$$

#### Variable-Temperature NMR Spectra

To monitor Equation (1) in solution further, the temperature dependence of ^1^H and ^19^F NMR resonances was examined. The spectra were taken at 20° intervals, from 25 °C to −60 °C, and are shown in Figures S3–S6. The temperature variation study was performed almost 60 days after the NMR spectra were first recorded for **2·H_2_O_(red)_** (Experimental section), when Pt^II^ and Pt^IV^ were present in an almost 1:1 ratio. The integration ratios of Pt^IV^ to Pt^II^ are 40% and 60%, respectively, at 25 °C.

^1^H and ^19^F NMR spectra do not show a any significant change in the temperature range of 25 °C to −60 °C. The F 2, 6 and F 3, 5 resonances for Pt^II^ do not show any change with variation in the temperature. However, a slightly increased separation between F 3, 5 resonances of Pt^II^ and Pt^IV^ was observed in the ^19^F NMR spectra, with some broadening and reduced definition of F 3, 5 of the Pt^IV^ species. The broadening is attributed to crystallization at lower temperatures. The integration ratio of Pt^IV^ to Pt^II^ decreases upon cooling, consistent with partial crystallization of the former.

When the solution was heated from 25 °C to 50 °C and the spectra were collected, the **1H** resonances showed an increase in intensity in the ^19^F NMR spectra consistent with the further extent of the reaction in Equation (1), as shown in Figure S6.

### 2.5. Electrospray MS Measurements

As described in the supporting information, no Pt^IV^ complexes were detected via electrospray MS measurements. Similar to what was observed for *trans*-organoenamineamidoplatinum(II) complexes [47], the starting material **1** was observed.

## 3. Materials and Methods

### 3.1. Chemicals

The solvents acetone, dichloromethane, acetonitrile, ethyl acetate, and *n*-hexane were HPLC grade. Hydrogen peroxide (30% solution in water) (Merck) was stored at −4°C. MnO_2_, NBu_4_Cl, and LiCl were from Sigma Aldrich and used as received. The *Class 2* organoamidoplatinum(II) complex, *trans*-[Pt{(*p*-BrC_6_F_4_)NCH_2_CH_2_NEt_2_}Cl(py)] (**1**), was synthesized by using the literature method **[62]**.

### 3.2. Instrumentation/Analytical Procedure

NMR spectra were recorded in deuterated solvents with Bruker DPX 300, 400, or 600 spectrometers (Billerica, MA, USA) supported by Top Spin NMR 4.3.0 software on a Windows NT workstation. CFCl_3_ and tetramethylsilane were used for the internal calibration of ^19^F NMR and ^1^H NMR spectra, respectively. Infrared spectra were recorded on a Perkin-Elmer 1600 FT-IR spectrophotometer as Nujol and hexachlorobutadiene (HCB) mulls between NaCl plates or recorded with an Agilent Cary 630 (Agilent Technologies Ltd., Yarnton, UK) attenuated total reflectance (ATR) spectrometer in the range 4000–600 cm^−1^. Low-resolution ESI measurements were recorded on a Waters micromass ZQ QMS connected to an Agilent 1200 series HPLC system. High-resolution accurate mass measurements were performed on a TOF (Agilent) instrument with a multimode source by using the dual methods ESI (electrospray ionization) and APCI (atmospheric pressure chemical ionization). Microanalyses were carried out by the Science Centre, London Metropolitan University Elemental Analysis Service. An electrothermal IA6304 apparatus was used to measure the melting points (uncalibrated) of the compounds. PXRD patterns were measured using a Bruker D8-Focus diffractometer (Billerica, MA, USA) with a 1° divergence slit, 0.2° receiving slit, and carbon monochromators (Cu-Kα radiation, λ = 1.5406 Å) in the range 2θ = 2–60° at 0.02° increments, at room temperature. The Mercury 4.3.0 software was used to generate the calculated powder patterns generated from the single-crystal diffraction models.

### 3.3. X-ray Crystallography

X-ray diffraction data obtained from single crystals of **2·H_2_O_(gold)_**, [NBu_4_][PtCl_3_(py)], and **3** were collected at a wavelength of λ = 0.712 Å using the MX1 beamline at the Australian Synchrotron, Victoria, Australia, with Blue Ice [72], a GUI using the same method as mentioned in the Experimental section of the previous report [62]. Data were processed with the XDS [73] version 20230630 software package. The structures were solved using direct methods with SHELXS-97 [74] and refined using conventional alternating least-squares methods with SHELXL-97 [74]. Single crystals of **2·H_2_O_(red)_** were loaded onto a fine glass fiber or cryoloop using hydrocarbon oil and the data collected at 123K using an open-flow N_2_ Oxford Cryptosystem. A Bruker Apex II diffractometer was used to collect the data, which was processed using the SAINT [75] program. The program *OLEX2* [76] was used as the graphical interface. All non-hydrogen atoms in the structures were refined anisotropically, and hydrogen atoms attached to carbon were placed in calculated positions and allowed to ride on the atom to which they were attached. The positions of the hydrogen atoms attached to the oxygen atoms were experimentally located and refined by using multiple refinement cycles.

Crystallographic data for all the structures reported in this paper have been deposited with the Cambridge Crystallographic Data Centre as supplementary number CCDC 2272032 for **2·H_2_O_(gold)_**, 2272033 for **2·H_2_O_(red)_**, 2272037 for **3**, and 2280550 for [NBu_4_][PtCl_3_(py)]. Copies of the data can be obtained free of charge at www.ccdc.cam.ac.uk/data_request/cif (accessed on 31 July 2023).

### 3.4. Experimental Section

#### 3.4.1. Oxidation of **1** with H_2_O_2_

In acetone: **1** (0.630 g, 1.0 mmol) was dissolved in 16 mL acetone, and (0.2 mL, 2.0 mmol) of 30% H_2_O_2_ solution was added. The reaction mixture was stirred at room temperature for 12 days. The color of the solution changed from initially yellow to deep red and then to reddish-orange; MnO_2_(see the warning below) [77] (2 g) was added at that time. After filtration and evaporation of the solution to 5–6 mL, distilled water (20 mL) was added, producing a cloudy solution with deep-red oil. The cloudy solution was decanted off, filtered, and a red–brown powder was collected. Crystallization of the red–brown powder from acetone/hexane produced crystals of **1H** by slow evaporation, characterized by X-ray single-crystal diffraction, ^1^H and ^19^F NMR, and mass spectrometry.

**(a) [Pt{(*p-*BrC_6_F_4_)NCH=CHNEt_2_}Cl(py)] (1H):** Metallic gold-colored blocks. (0.002 g, 1.4% crystal yield). ^19^F NMR (CD_3_(CO)_2_): −148.2 [m, 2 F, F 2, 6], −138.2 [m, 2 F, F 3,5]. ^1^H NMR (CD_3_(CO)_2_): 1.56 [td, 6 H, ^3^J_H,H_ 7 Hz, NCH_2_C**H_3,_**], 2.30 [m, 2 H, NC**H_A_**H_B_CH_3,_], 3.43 [m, 2 H, NC**H_B_**H_A_CH_3,_], 3.75 [d, ^3^J_H,H_ 3.45 Hz, ^3^J_H,Pt_ 34 Hz, 1 H, C**H**NEt_2_], 6.07 [m with ^195^Pt satellites, ^3^J_H,Pt_ 50 Hz, 1 H, C**H**N(*p-*BrC_6_F_4_)], 7.19 [m, 2 H, **H 3**, **5** (py)], 7.74 [tt, ^3^J_H,H_ 7.8 Hz, ^4^J_H,H_ 1 Hz, 1 H, **H 4** (py)], 8.42 [d with ^195^Pt satellites, ^3^J_H,H_ 5.6 Hz, ^3^J_H,Pt_ 30 Hz, 2 H, **H 2**, **6** (py)]. All these data (IR and ESI *m/z* (+ve) are given in SI) agree withthose reported for **1H** [47].

The remaining red–orange filtrate was concentrated using a rotatory evaporator. The entire solution changed color from reddish-orange to gold, and more red–brown oil was obtained. After separation from the oil, the gold-colored filtrate produced shiny golden flakes upon cooling at −10 °C. These golden flakes were recrystallized from acetone/water, and gold-colored crystals of the platinum(IV) complex **2·H_2_O(H_2_O)** were obtained. After collecting crystals, slow evaporation of the remaining mother liquor produced [Pt{(*p-*BrC_6_F_4_)NCH=C(H_0.25_Br_0.75_)NEt_2_}Cl(py)], **1H_0.25_Br_0.75_**, as characterized by X-ray crystallography, yielding the same unit cell as reported earlier [47]. The red–brown oil was dissolved in acetone, and many unsuccessful attempts of crystallization were made with various solvents. ^1^H and ^19^F NMR revealed the presence of Pt^IV^ species, **2·H_2_O(H_2_O)**, with some Pt(py)_2_Cl_2_. Some colorless shiny crystals of trans-Pt(py)_2_Cl_2_ were obtained after a long period and were characterized by X-ray diffraction.

**(b) *cis, cis, trans-* [Pt{(*p-*BrC_6_F_4_)NCH_2_CH_2_NEt_2_}Cl(py)(OH)_2_].H_2_O 2·H_2_O_(gold)_:** Metallic gold blocks. (0.160 g, 25% yield). M.P. = 192 °C (dec.). Elemental analysis calcd for C_17_H_23_Cl_1_F_4_N_3_Pt_1_Br_1_O_3_ (M = 702.02): C, 29.03%; H, 3.30%; N, 6.04%. Found: C, 29.20%; H, 3.17%; N, 6.08%.

*In solution, **2·H_2_O_(gold)_** yielded **1H**:

^19^F NMR, ^1^H NMR, and MS data agree with those of **1H** above.

#### 3.4.2. Oxidation of **1** with 30% H_2_O_2_ in the Presence of LiCl

**1** (0.325 g, 0.5 mmol) was dissolved in 15 mL CH_2_Cl_2_ and lithium chloride (0.021 g, 0.50 mmol), and a 30% solution of H_2_O_2_ (0.1 mL, 1 mmol) was added. The reaction mixture was stirred at room temperature for 7 days. The color of the solution changed from initial orange to deep red after 2 days and then to orange–red. MnO_2_ (2 g) was added, the solution was stirred for 0.5 h, filtered, and MnO_2_ was washed with acetone. After concentrating the filtrate, distilled water (6 mL) was added until it became cloudy. A deep-red-colored oil formed with a cloudy solution. The red oil was separated and dissolved in acetone, and crystallization from acetone/hexane at −10 °C produced deep-red-colored blocks of the platinum(IV) species, **2·H_2_O_(red)_** (0.1637 g, yield = 50%). The cloudy solution was evaporated until dryness and then dissolved again in acetone; crystallization from acetone/hexane produced the mononuclear platinum(IV) species golden **2·H_2_O** in a 14% yield, identified by X-ray crystallography.

WARNING: Concentrated H_2_O_2_ in the acetone in presence of an acid catalyst can form the shock and friction-sensitive explosive triacetone triperoxide (TATP). MnO_2_ was used to decompose any residual H_2_O_2_ catalytically before workup [77].

**(a) *cis, cis, trans-* [Pt{(*p-*BrC_6_F_4_)NCH_2_CH_2_NEt_2_}Cl(py)(OH)_2_].H_2_O 2·H_2_O_(red)_:** Metallic deep-red-colored blocks. (0.164 g, 50% yield). M.P. = 172 °C. (dec). Elemental analysis calcd for C_17_H_23_Br_1_Cl_1_F_4_N_3_Pt_1_O_3_ (M = 702.2): C, 29.01%; H, 3.29%; N, 5.97%. Found: C, 29.28%; H, 3.16%; N, 6.03%.

*Both Pt^II^ **(1H)** and Pt^IV^ **2** were observed in the solution in a 1:1 ratio.

^19^F NMR (CD_3_(CO)_2_): **Pt^II^**, **1H:** −148.16 [m, 2 F, F 2,6], −138.24 [m, 2 F, F 3,5]; **Pt^IV^**, **2:** −138.34 [m, 2 F, F 3,5], −140.64 [m, 2 F, F 2, 6].^1^H NMR (CD_3_(CO)_2_): 1.23 [t, 6H, ^3^J_H,H_ 7 Hz, ^4^J_H,H_ 3Hz, NCH_2_C**H_3_** (**2**)], 1.56 [t, 6H, ^3^J_H,H_ 7 Hz, ^4^J_H,H_ 3Hz, NCH_2_C**H_3_** (**1H**)], 2.30 [m, 2H, C**H_2_**NEt_2_ (**2**)], 2.77 [m, 2H, NC**H_A_**H_B_CH_3_ (**1H**)], 3.00 [m, 2H, NC**H_A_**H_B_CH_3,_ (**2**)], 3.13 [m, 2H, NC**H_B_**H_A_CH_3_ (**2**)], 3.33 [m, 2H, C**H_2_**N(*p-*BrC_6_F_4_) (**2**)], 3.43 [m, 2H, NC**H_B_**H_A_CH_3_ (**1H**)], 3.75 [d with ^195^Pt satellites, ^3^J_H,H_ 3.45 Hz, ^3^J_H,Pt_ 34 Hz, 1H, C**H**NEt_2_ (**1H**)], 4.03 [m, 2H, not exchangeable with D_2_O], 6.07 [m with ^195^Pt satellites, ^3^J_H,Pt_ 50 Hz, 1H, C**H**N(*p-*BrC_6_F_4_) (**1H**)], 7.19 [m, 2H, **H3**,**5** (py)], (**1H**)] 7.33 [t, ^3^J_H,H_ 7 Hz, 2H, **H3**,**5** (py) (**2**)]], 7.74 [tt,^3^J_H,H_ 7.8 Hz, ^4^J_H,H_ 1Hz, 1H, **H4** (py) (**1H**)], 7.86 [t, ^3^J_H,H_ 7.7 Hz, 1H, **H4** (py) (**2**)], 8.42 [d with ^195^Pt satellites, ^3^J_H,H_ 5.6 Hz, ^3^J_H,Pt_ 35 Hz, 2H, **H2**,**6** (py) (**1H**)], 8.98 [d with Pt satellites, ^3^J_H,H_ 5.6 Hz, ^3^J_H,Pt_ 35 Hz, 2H, **H 2**,**6** (py) (**2**)]. IR: 3549m, 3050w, 2964w, 2929w, 2871w, 2372w, 2108w, 1922w, 1704w, 1655s, 1620s, 1475s, 1451w, 1374m, 1287w, 1261w, 1223m, 1141s, 1061m, 1014w, 968s, 917m, 834w, 820s, 763s, 741s, 694s, 638w cm^−1^. (ESI *m/z* (+ve) are given in SI).

#### 3.4.3. Oxidation of **1** with Excess H_2_O_2_ in the Presence of Tetrabutylammonium Chloride (TBACl)

In a solution of **1** (0.314 g, 0.48 mmol) in 20 mL CH_2_Cl_2_, 0.127 g TBACl (0.48 mmol) in 2 mL of CH_2_Cl_2_ and (0.1 mL, 1.0 mmol) of 30% solution of H_2_O_2_ were added. The solution was heated at near refluxing temperature for 6 h and then stirred at room temperature for 4 days. The color of the solution changed from yellow to dark orange, but not deep red; MnO_2_ was added at this stage. After filtration, the solution was concentrated to 5 mL, and a deep-red-colored oil formed when hexane (6 mL) was added. This mixture was cooled at −10 °C. Deep-red-colored blocks of the platinum(IV) complex **3** were collected by filtration and then air-dried. Some **2·H_2_O_(gold)_** was collected from the filtrate with a few pale-yellow crystals of [NBu_4_][PtCl_3_(py)] characterized by single-crystal X-ray crystallography only.

**(a) *cis, cis, trans-* [{Pt{(*p-*BrC_6_F_4_)NCH_2_CH_2_NEt_2_}Cl(py)(OH)_2_}_2_].CH_2_Cl_2_, 3·0.5CH_2_Cl_2_:** Metallic red-colored blocks. (0.152.9g, 49% crystal yield). Elemental analysis calcd for C_35_H_44_Cl_4_F_8_N_6_Pt_2_Br_2_O_4_ (M = 1456.56): C, 28.86%; H, 3.04%; N, 5.77%. Found: C, 29.05%; H, 3.13%; N, 5.65%.

*In the solution Pt^II^ (**1H** and **1Cl**) and Pt^IV^ are present. The ratio of platinum(IV), **1H,** and **1Cl** is 1:2:0.7, respectively. Most of the NMR resonances of **1H** and **1Cl** are overlapped.

^19^F NMR (CD_3_(CO)_2_): **1H**: −148.61 [m, 2 F, F 2, 6], −137.96 [m, 2 F, F 3,5]; **1Cl**: −148.46 [m, 2 F, F 2, 6], −137.27 [m, 2 F, F 3,5]; **Pt^IV^**,(**3**): −137.63 [m, 2 F, F 3,5], −142.25 [m, 2 F, F 2,6]. ^1^H NMR (CD_3_(CO)_2_): 1.26 [t, 6H, ^3^J_H,H_ 7 Hz, ^4^J_H,H_ 3Hz, NCH_2_C**H_3,_** (**3**)], 1.61 [t, 6H, ^3^J_H,H_ 7 Hz, ^4^J_H,H_ 3Hz, NCH_2_C**H_3_**, (**1H**+**1Cl**)], 2.30 [m, 2H, C**H_2_**NEt_2,_ (**3**)], 2.70 [m, 2H, NC**H_A_**H_B_CH_3_, (**1H**+**1Cl**)], 3.00 [m, 2H, NC**H_A_**H_B_CH_3_ and 2H, NC**H_B_**H_A_CH_3,_(**3**)], 3.47 [m, 2H, NC**H_B_**H_A_CH_3_ and 2H, C**H_2_**N(*p-*BrC_6_F_4_), (**3**)], 3.68 [d with ^195^Pt satellites, ^3^J_H,H_ 3.45 Hz, ^3^J_H,Pt_ 34 Hz, 1H, C**H**NEt_2_, **(1H)**], 4.03 [m, 2H, not exchangeable with D_2_O], 6.01 [m with ^195^Pt satellites, ^3^J_H,Pt_ 50 Hz, 1H, C**H**N(*p-*BrC_6_F_4_), **(1H)**], 6.43 [t, 0.5 H, C**H**NEt_2_, **(1Cl)**], 6.47 [t, 0.5 H, C**H**NEt_2_, **(1Cl)**], 7.04 [m, 2H, **H 3**, **5** (py), (**1H**+**1Cl**)], 7.19 [t, ^3^J_H,H_ 7 Hz, 2H, **H 3**, **5** (py), (**3**)], 7.59 [tt,^3^J_H,H_ 7.8 Hz, ^4^J_H,H_ 1Hz, 1H, **H4** (py), (**1H**+**1Cl**)], 7.72 [t, ^3^J_H,H_ 7.7 Hz, 1H, **H4** (py), (**3**)], 8.39 [with ^195^Pt satellites, ^3^J_H,H_ 5.6 Hz, ^3^J_H,Pt_ 35 Hz, 2H, **H2**,**6** (py), (**1H**+**1Cl**)], 8.98 [d with ^195^Pt satellites, ^3^J_H,H_ 5.6 Hz, ^3^J_H,Pt_ 35 Hz, 2H, **H2**,**6** (py), (**3**)]. IR: 3652m, 3388br, 2963w, 2875w, 1923w, 1704w, 1655s, 1620s, 1475s, 1451w, 1374m, 1287w, 1261w, 1223m, 1141s, 1061m, 1014w, 968s, 917m, 834w, 820s, 763s, 741s, 694s, 638w cm^−1^.

The data for **1H** and **1Cl** agree with that reported [47].

## 4. Conclusions

The oxidation of [Pt{(*p*-BrC_6_F_4_)NCH_2_CH_2_NEt_2_}Cl(py)], **1** by limited H_2_O_2_ at room temperature produced dihydroxidoplatinum(IV) complexes [Pt^IV^{(*p*-BrC_6_F_4_)NCH_2_CH_2_NEt_2_}Cl(OH)_2_(py)].H_2_O, **2·H_2_O**, and [Pt^IV^{(*p*-BrC_6_F_4_)NCH_2_CH_2_NEt_2_}Cl(OH)_2_(py)].0.5CH_2_Cl_2_, **3·0.5CH_2_Cl_2_**, depending on the solvent system used. **2·H_2_O** was obtained as two different colored crystals, red and gold. Identical unit cells and X-ray powder diffraction patterns generated from their X-ray crystal structures verify that they have the same structure. As the initial preparation of **2·H_2_O_(gold)_** was accompanied by the formation of the Pt^II^ complex with the oxidized ligand [Pt{(*p*-BrC_6_F_4_)NCH=CHNEt_2_}Cl(py)] **1H**, different conditions, including changes in solvents and the addition of chloride salts, were used to make the reactions more selective and produce higher yields in preparations of **2·H_2_O_(red)_** and **3·0.5CH_2_Cl_2_**. These Pt^IV^ compounds show reductive dehydration, that is, reduction of Pt^IV^ to Pt^II^, accompanied by oxidation of the ligand over time in the solid state, as determined by X-ray powder diffraction and the separation of crystals, and in solution as evident from NMR and Mass spectra. The main product is **1H,** but co-crystallized **1H/1Cl** (**1Cl** = [Pt(*p*-BrC_6_F_4_)NCH=C(Cl)NEt_2_}Cl(py)]) was obtained in some cases. This reveals that the Pt^IV^ complexes are precursors in the formation of the oxidized ligand species **1H** and **1Cl**. The rate of reductive dehydration is faster for **2·H_2_O_(gold)_** than for **2·H_2_O_(red)_**. Vigorous oxidation conditions lead to complexes of the oxidized ligand [47], whereas the mild conditions currently used provide access to Pt^IV^. As a wide range of *Class* 2 organoamidoplatinum(II) compounds are known [46], access to many derived Pt^IV^ compounds is possible, and some of which may have greater stability than **2·H_2_O** and **3·0.5CH_2_Cl_2_**.

## Figures and Tables

**Table 1:** Quantities of reagents and product yields (crystalline) for the oxidation of **1** with 30% H_2_O_2_ reactions performed at room temperature.

Compound	Cl Source	H_2_O_2_	Solvent	Time	Products with Yields
**1** **(1.0 mmol)**	none	2.0 mmol	CH_3_COCH_3_	12 d	**1H** = 1.4%, **2·H_2_O_(gold)_** = 25%; **1H_0.25_1Br_0.75_** = 7%
**1** **(0.5 mmol)**	LiCl (0.5 mmol)	1.0 mmol	* CH_2_Cl_2_	7 d	**2·H_2_O_(red)_** = 50%, **2·H_2_O_(gold)_** = 14%
**1** **(0.48 mmol)**	NBu_4_Cl (0.48 mmol)	1.0 mmol	* CH_2_Cl_2_	4 d	**3·0.5CH_2_Cl_2_** = 49%, **2·H_2_O_(gold)_** = 10%

* workup differs (see experimental).

**Table 2:** Crystallographic data for the molecular structures of **2·H_2_O_(gold)_**, **2·H_2_O_(red)_**, and **3·0.5CH_2_Cl_2_**.

	2·H_2_O_(gold)_	2·H_2_O_(red)_	3·0.5CH_2_Cl_2_
Empirical formula	C_17_H_19_BrClF_4_N_3_O_3_Pt	C_17_H_19_BrClF_4_N_3_O_3_Pt	C_17.5_H_22_BrCl_2_F_4_N_3_O_2_Pt
Formula weight	703.83	703.83	728.28
Crystal system	Monoclinic	Monoclinic	Monoclinic
Space group	*C*2/*c*	*Cc*	*C*2/*c*
*a* (Å)	19.719(4)	19.6389(16)	14.948(3)
*b* (Å)	13.357(3)	13.3360(11)	14.302(3)
*c* (Å)	16.946(3)	16.9327(13)	21.743(4)
*α* (°)	90	90	90
*β* (°)	105.29(3)	104.857(2)	104.53(3)
*γ* (°)	90	90	90
vol (Å^3^)	4305.4(16)	4286.5(6)	4499.7(17)
*Z*	8	8	8
*ρ* (calcd) (g/cm^3^)	2.172	2.169	2.150
*µ* (mm^−1^)	8.557	8.594	8.303
F (000)	2688.0	2656.0	2776.0
Reflections collected/unique	31,692/5071	35,311/12,158	40,998/6383
*R_int_*	0.1016	0.0537	0.0504
2*θ*_max_ (°)	56.33	61.2	63.5
Goodness-of-fit on F^2^	1.287	1.044	1.060
*R1* indices [I ≥ 2σ (I)]	0.0712	0.0387	0.0342
*wR2* indices [I ≥ 2σ (I)]	0.1431	0.0634	0.0841
Flack parameter	n/a	0.398(5)	n/a

n/a = not applicable.

**Table 3:** Selected bond lengths for compounds **2·H_2_O_(gold)_**, **2·H_2_O_(red)_** (given for both molecules in asymmetric units), and **3·0.5CH_2_Cl_2_**.

Bond	2·H_2_O_(gold)_ C_17_H_23_BrClF_4_N_3_O_3_Pt (Å)	2·H_2_O_(red)_ C_17_H_23_BrClF_4_N_3_O_3_Pt (Molecules A and B in Asymmetric Unit) (Å)		3·0.5CH_2_Cl_2_ C_17.5_H_22_BrCl_2_F_4_N_3_O_2_Pt (Å)	103(OH)_2_ C_24_H_18_F_8_N_4_O_2_Pt [54] (Å)
Pt-O1	2.029(9)	2.015(6)	2.028(7)	2.061(3)	2.017(3)
Pt-O2	2.003(8)	1.999(6)	2.016(6)	2.018(3)	2.008(3)
Pt-Cl	2.349(3)	2.359(3)	2.345(3)	2.3662 (11)	n/a
Pt-N1_(amide)_	2.031(9)	2.026(9)	2.037(9)	2.047(3)	2.033(3)
Pt-N2_(amine)_	2.119(7)	2.119(8)	2.132(9)	2.124(3)	n/a
Pt-N3_(py)_	2.078(7)	2.087(8)	2.066(9)	2.061(3)	2.083(3)
N1_(amide)_-C_6_F_4_	1.395(13)	1.387(15)	1.380(14)	1.391(5)	1.392(5)
C7_(deen)_-C8_(deen)_	1.492(16)	1.507(15)	1.497(15)	1.506(6)	1.473(5)
N1_(amide)_-C7_(deen)_	1.459(12)	1.456(13)	1.469(13)	1.467 (5)	1.470(5)
N2_(amine)_-C8_(deen)_	1.508(14)	1.497(13)	1.504(13)	1.510(6)	n/a
N2_(amine)-_C9_(Et)_ N2_(amine)-_C11_(Et)_	1.510(14) 1.502(13)	1.510(13) 1.508(14)	1.514(14) 1.508(13)	1.497(5) 1.521(5)	n/a

n/a = not applicable.

**Table 4:** Inter- and intramolecular H-bonding interaction distances for **2·H_2_O_(gold)_** and **3·0.5CH_2_Cl_2_**.

2·H_2_O_(gold)_		3·0.5CH_2_Cl_2_	
Interactions	Distance	Interactions	Distance
F1⋯HO2	2.412(7)	O2⋯ H(CH_2_CH_3_)	2.3561(8)
F4⋯ HO1	2.438(7)	F4⋯HO2	2.7065(7)
O1⋯ H(py)	2.322(7)	Cl⋯H(CH_3_)	2.7529(7)
O2⋯ H(py)	2.227(7)	(CH_2_Cl_2_)Cl⋯H(CH_2_N(*p*-BrC_6_F_4_)	3.0946(7)
O1⋯ H(CH_2_CH_3_)	2.380(7)		
Cl⋯H(CH_3_)	2.738(3)	Inter (CH_2_Cl_2_)Cl⋯H(CH_2_CH_3_)	2.8282(4)
Inter O3H⋯O1	2.198(7)		

**Table 5:** ^1^H and ^19^F NMR chemical shifts and assignments for **1H**, **2·H_2_O_(red)_**, and **3·0.5CH_2_Cl_2_** in (CD_3_)_2_CO.

^1^H NMR Chemical Shifts Assignment	1H * (ppm)	2·H_2_O_(red)_ (ppm)	3·0.5CH_2_Cl_2_ (ppm)
-NCH_2_C**H_3_**	1.56, td	1.23, t	1.26, t
-C**H_2_**NEt_2_	-	2.30, m	2.30, m
=C**H**NEt_2_	3.75, d	-	-
-NC**H_A_**H_B_CH_3_	2.30, m	3.00, m	3.00, m
-NCH_A_**H_B_**CH_3_	3.43, m	3.13, m	3.00, m
-C**H_2_**N(*p-*BrC_6_F_4_)	-	3.33, m	3.47, m
=CHN(*p-*BrC_6_F_4_)	6.07, m	-	-
Pt-OH	-	4.03, m	4.03, m
H 3, 5 (py)	7.19, m	7.33, t	7.19, t
H 4 (py)	7.74, tt	7.86, t	7.72, t
H 2,6 (py)	8.42, d	8.98, d	8.98, d
^19^F NMR chemical shifts Assignment	1H * (ppm)	**2·H_2_O_(red)_** (ppm)	**3·0.5CH_2_Cl_2_** (ppm)
(*p-*BrC_6_F_4_) F 3,5	−138.20, m	−138.34, m	−137.63, m
(*p-*BrC_6_F_4_) **F** 2,6	−148.20, m	−140.64, m	−142.25, m

The data are extracted from complex NMR spectra of the compounds plus their reductive dehydration products. The full NMR data and integrations showing relative amounts are provided in the Experimental section. * For **2·H_2_O_(gold)_**_,_ only **1H** was observed. Pt^IV^ was not observed.

## Data Availability

Crystal data can be obtained from Cambridge Crystallographic Data Centre as supplementary number CCDC 2272032 for **2·H_2_O_(gold)_**, 2272033 for **2·H_2_O_(red)_**, 2272037 for **3**, and 2280550 for [NBu_4_][PtCl_3_(py)]. Copies of the data can be obtained free of charge at www.ccdc.cam.ac.uk/data_request/cif (accessed on 31 July 2023).

## Supplementary Materials

The following supporting information can be downloaded at: https://www.mdpi.com/article/10.3390/molecules28176402/s1, **S1. Pt^IV^ data [Table S1**: Selected bond angles for compounds **2·H_2_O_(gold)_** and **3·0.5CH_2_Cl_2_**; Figure S1: Comparison of Powder XRD patterns calculated from single-crystal X-ray diffraction data of gold and **2**·**H_2_O_(red)_** presented in the respective colors. Table S2: Selected bond angles for compound **2·H_2_O_(red)_**.**] S2. [NBu_4_][PtCl_3_(py)] Data [**Figure S2: Molecular structure of [NBu_4_][PtCl_3_(py)], showing 50% thermal ellipsoids. Table S3: Crystallographic data for the molecular structures of [NBu_4_][PtCl_3_(py)]; Table S4: Selected bond lengths and bond angles of [NBu_4_][PtCl_3_(py)].**] S3. Variable temperature NMR spectra [**Figure S3: ^19^F NMR spectra obtained for **2·H_2_O_(red)_** over the temperature range of 25 °C to −60 °C. Figure S4: ^1^H NMR spectra obtained for **2·H_2_O_(red)_** over the temperature range of 25 °C to −60 °C, showing 6–9 ppm region. Figure S5: ^1^H NMR spectra obtained for **2·H_2_O_(red)_** over the temperature range from 25 °C to −60 °C, showing 1–4.5 ppm region. Figure S6: ^19^F NMR spectra obtained for **2·H_2_O_(red)_** at 25°C and 50°C.**] S4. PXRD data [**Figure S7: Normalized powder X-ray diffraction data for bulk samples of **2·H_2_O_(gold)_** and **2·H_2_O_(red)_**, shown in their respective colors. Figure S8: Normalized powder X-ray diffraction data for bulk sample of **2·H_2_O_(red)_** with normalized powder X-ray diffraction data generated from the single crystal of **1H** and **2·H_2_O_(red)_**; Figure S9: Normalized powder X-ray diffraction data for bulk sample of **2·H_2_O_(gold)_** with normalized powder X-ray diffraction data generated from the single crystals of **1H** and **2·H_2_O_(gold)_ [78,79,80,81]**.**] S5. Isolation of Pt^IV^ from the solution of an aged bulk sample [**Table S5 Unit cell parameters for **1H** and co-crystallized **1(H/Cl)** collected from the bulk sample of **2·H_2_O_(red)_** and **2·H_2_O_(gold)_. Table S6**: Unit cell parameters for Pt^IV^ **2·H_2_O_(red)ʹ_,** co-crystallized **1(H/Cl)** and [NBu_4_][PtCl_3_(py)] isolated from the solution of an aged sample of **3·0.5CH_2_Cl_2_. S6. Electrospray MS measurements. S7. IR Spectroscopy data.**

## Sample Availability

Samples of the compounds are available from the authors.

## References

[1] Rosenberg B., Van Camp L., Krigas T. (1965). Inhibition of cell division in escherichia coli by electrolysis products from a platinum electrode. Nature.

[2] Johnstone T.C., Park G.Y., Lippard S.J. (2014). Understanding and improving platinum anticancer drugs—Phenanthriplatin. Anticancer Res..

[3] Wong E., Giandomenico C.M. (1999). Current status of platinum-based antitumor drugs. Chem. Rev..

[4] Wang D., Lippard S.J. (2005). Cellular processing of platinum anticancer drugs. Nat. Rev. Drug Discov..

[5] Kelland L. (2007). The resurgence of platinum-based cancer chemotherapy. Nat. Rev. Cancer.

[6] Fricker S.P. (2007). Metal based drugs: From serendipity to design. Dalton Trans..

[7] Wheate N.J., Walker S., Craig G.E., Oun R. (2010). The status of platinum anticancer drugs in the clinic and in clinical trials. Dalton Trans..

[8] Farrell N.P. (2011). Platinum formulations as anticancer drugs clinical and pre-clinical studies. Curr. Top. Med. Chem..

[9] Stewart D.J. (2007). Mechanisms of resistance to cisplatin and carboplatin. Crit. Rev. Oncol. Hematol..

[10] Rabik C.A., Dolan M.E. (2007). Molecular mechanisms of resistance and toxicity associated with platinating agents. Cancer Treat. Rev..

[11] Weiss R.B., Christian M.C. (1993). New cisplatin analogs in development—A Review. Drugs.

[12] Hartmann J.T., Lipp H.P. (2003). Toxicity of platinum compounds. Expert Opin. Pharmacother..

[13] Abu-Surrah A.S., Kettunen M. (2006). Platinum group antitumor chemistry: Design and development of new anticancer drugs complementary to cisplatin. Curr. Med. Chem..

[14] Todd R.C., Lippard S.J. (2009). Inhibition of transcription by platinum antitumor compounds. Metallomics.

[15] Klein A.V., Hambley T.W. (2009). Platinum drug distribution in cancer cells and tumors. Chem. Rev..

[16] Piccart M., Lamb H., Vermorken J.B. (2001). Current and future potential roles of the platinum drugs in the treatment of ovarian cancer. Ann. Oncol..

[17] Hambley T.W. (1997). The influence of structure on the activity and toxicity of Pt anticancer drugs. Coord. Chem. Rev..

[18] Gibson D. (2016). Platinum(iv) anticancer prodrugs—Hypotheses and facts. Dalton Trans..

[19] Lee Y.-A., Lee S.S., Kim K.M., Lee C.O., Sohn Y.S. (2000). Synthesis and oral antitumor activity of tetrakis(carboxylato)platinum(IV) complexes. J. Med. Chem..

[20] Barbanente A., Gandin V., Ceresa C., Marzano C., Ditaranto N., Hoeschele J.D., Natile G., Arnesano F., Pacifico C., Intini F.P. (2022). Improvement of Kiteplatin efficacy by a benzoato Pt(IV) prodrug suitable for oral administration. Int. J. Mol. Sci..

[21] Galanski M., Keppler B.K. (2007). Searching for the magic bullet: Anticancer platinum drugs which can be accumulated or activated in the tumor tissue. Anticancer Agents Med. Chem..

[22] Hall M.D., Mellor H.R., Callaghan R., Hambley T.W. (2007). Basis for design and development of platinum(IV) anticancer complexes. J. Med. Chem..

[23] Harrap K.R., Kelland L.R., Jones M., Goddard P.M., Orr R.M., Morgan S.E., Murrer B.A., Abrams M.J., Giandomenico C.M., Cobbleigh T. (1991). Platinum coordination complexes which circumvent cisplatin resistance. Adv. Enzym. Res..

[24] Albert A. (1958). Chemical aspects of selective toxicity. Nature.

[25] Xiao H., Song H., Yang Q., Cai H., Qi R., Yan L., Liu S., Zheng Y., Huang Y., Liu T. (2012). A prodrug strategy to deliver cisplatin(IV) and paclitaxel in nanomicelles to improve efficacy and tolerance. Biomaterials.

[26] Hall M.D., Hambley T.W. (2002). Platinum(IV) antitumour compounds: Their bioinorganic chemistry. Coord. Chem. Rev..

[27] Yao H., Wang Z., Wang N., Deng Z., Liu G., Zhou J., Chen S., Shi J., Zhu G. (2022). Enhancing circulation and tumor accumulation of carboplatin via an erythrocyte-anchored prodrug strategy. Angew. Chem. Int. Ed..

[28] Ellis L.T., Er H.M., Hambley T.W. (1995). The influence of the axial ligands of a series of platinum(IV) anticancer complexes on their reduction to platinum(II) and reaction with DNA. Aust. J. Chem..

[29] Chaney S.G., Wyrick S., Till G.K. (1990). In vitro biotransformations of tetrachloro(d,l-trans)-1,2-diaminocyclohexaneplatinum(IV) (tetraplatin) in rat plasma. Cancer Res..

[30] Poon G.K., Raynaud F.I., Mistry P., Odell D.E., Kelland L.R., Harrap K.R., Barnard C.F., Murrer B.A. (1995). Metabolic studies of an orally active platinum anticancer drug by liquid chromatography-electrospray ionization-mass spectrometry. J. Chromatogr..

[31] Choi S., Filloto C., Bisanzo M., Delaney S. (1998). Reduction and anticancer activity of Platinum(IV) complexes. Inorg. Chem..

[32] Huang J., Ding W., Zhu X., Li B., Zeng F., Wu K., Wu X., Wang F. (2022). Ligand evolution in the photoactivatable platinum(IV) anticancer prodrugs. Front. Chem..

[33] Spector D., Pavlov K., Beloglazkina E., Krasnovskaya O. (2022). Recent advances in light-controlled activation of Pt(IV) prodrugs. Int. J. Mol. Sci..

[34] Chen Q., Yang Y., Lin X., Ma W., Chen G., Li W., Wang X., Yu Z. (2018). Platinum(iv) prodrugs with long lipid chains for drug delivery and overcoming cisplatin resistance. Chem. Comm..

[35] Xie P., Jin Q., Li Y., Zhang J., Kang X., Zhu J., Mao X., Cao P., Liu C. (2022). Nanoparticle delivery of a triple-action Pt(IV) prodrug to overcome cisplatin resistance via synergistic effect. Biomater. Sci..

[36] Gibson D. (2021). Platinum(IV) anticancer agents; are we en route to the holy grail or to a dead end?. J. Inorg. Biochem..

[37] Sinisi M., Intini F.P., Natile G. (2012). Dependence of the reduction products of platinum(IV) prodrugs upon the configuration of the substrate, bulk of the carrier ligands, and nature of the reducing agent. Inorg. Chem..

[38] Nemirovski A., Vinograd I., Takrouri K., Mijovilovich A., Rompel A., Gibson D. (2010). New reduction pathways for ctc-[PtCl_2_(CH_3_CO_2_)_2_(NH_3_)(Am)] anticancer prodrugs. Chem. Commun..

[39] Hambley T.W. (1991). New approaches to platinum anti-cancer drugs. Chem. Aust..

[40] Lovejoy K.S., Lippard S.J. (2009). Non-traditional platinum compounds for improved accumulation, oral bioavailability, and tumor targeting. Dalton Trans..

[41] Cleare M.J., Hoeschele J.D. (1973). Studies on the antitumor activity of group VIII transition metal complexes. Part I. Platinum (II) complexes. Bioinorg. Chem..

[42] Farrell N. (2004). Metal Ions in Biological Systems.

[43] Wheate N.J., Collins J.G. (2005). Multi-nuclear platinum drugs: A new paradigm in chemotherapy. Curr. Med. Chem. Anticancer Agents.

[44] Wheate N.J., Collins J.G. (2003). Multi-nuclear platinum complexes as anticancer drugs. Coord. Chem. Rev..

[45] Webster L.K., Deacon G.B., Buxton D.P., Hillcoat B.L., James A.M., Roos I.A.G., Thomson R.J., Wakelin L.P.G., Williams T.L. (1992). Cis-bis(pyridine)platinum(II) organoamides with unexpected growth-inhibition properties and antitumor-activity. J. Med. Chem..

[46] Talarico T., Phillips D.R., Deacon G.B., Rainone S., Webster L.K. (1999). Activity and DNA binding of new organoamidoplatinum (II) complexes. Investig. New Drugs.

[47] Ojha R., Mason D., Forsyth C.M., Deacon G.B., Junk P.C., Bond A.M. (2021). Diverse and unexpected outcomes from oxidation of the platinum(II) anticancer agent [Pt{(*p*-BrC_6_F_4_)NCH_2_CH_2_NEt_2_}Cl(py)] by hydrogen peroxide. J. Inorg. Biochem..

[48] Masztafiak J., Nogueira J., Lipiec L., Kwiatek W., Wood B., Deacon G.B., Kayser Y., Fernandes D., Pavliuk M., Szlachetko J. (2017). Direct determination of metal complexes interation with DNA by atomic telemetry and multiscale molecular dynamics. J. Phys. Chem. Lett..

[49] Shaw P.A., Clarkson G.J., Rourke J.P. (2016). Long-lived five-coordinate platinum(IV) intermediates: Regiospecific c–c coupling. Organometallics.

[50] Twigg M.V. (2012). Higher Oxidation State Organopalladium and Platinum Chemistry. Platin. Met. Rev..

[51] Canty A.J., Hoare J.L., Davies N.W., Traill P.R. (1998). Synthesis and decomposition behavior of Pallada(IV)cyclopentane complexes. Organometallics.

[52] Canty A.J. (1992). Development of organopalladium(IV) chemistry: Fundamental aspects and systems for studies of mechanism in organometallic chemistry and catalysis. Acc. Chem. Res..

[53] Topczewski J.J., Sanford M.S. (2015). Carbon–hydrogen (C–H) bond activation at Pd(IV): A frontier in c–h functionalization catalysis. Chem. Sci..

[54] Guo S.-X., Mason D.N., Turland S.A., Lawrenz E.T., Kelly L.C., Fallon G.D., Gatehouse B.M., Bond A.M., Deacon G.B., Battle A.R. (2012). Systematic differences in electrochemical reduction of the structurally characterized anticancer platinum(IV) complexes [Pt{((*p*-HC_6_F_4_)NCH_2_)_2_}-(pyridine)_2_Cl_2_], [Pt{((*p*-HC_6_F_4_)NCH_2_)_2_}(pyridine)_2_(OH)_2_], and [Pt{((*p*-HC_6_F_4_)NCH_2_)_2_}(pyridine)_2_(OH)Cl]. J. Inorg. Biochem..

[55] Taylor R.A., Law D.J., Sunley G.J., White A.J.P., Britovsek G.J.P. (2008). Hydrogen bonding directs the H_2_O_2_ oxidation of platinum(ii) to a cis-dihydroxo platinum(iv) complex. Chem. Commun..

[56] Shamsuddin S., Santillan C.C., Stark J.L., Whitmire K.H., Siddik Z.H., Khokhar A.R. (1998). Synthesis, characterization, and antitumor activity of new platinum(IV) trans-carboxylate complexes: Crystal structure of [Pt(cis-1,4-DACH)trans-(acetate)2Cl2]. J. Inorg. Biochem..

[57] Lee Y.-A., Ho Yoo K., Jung O.-S. (2003). Oxidation of Pt(II) to Pt(IV) complex with hydrogen peroxide in glycols. Inorg. Chem. Commun..

[58] Canil G., Braccini S., Marzo T., Marchetti L., Pratesi A., Biver T., Funaioli T., Chiellini F., Hoeschele J.D., Gabbiani C. (2019). Photocytotoxic Pt(iv) complexes as prospective anticancer agents. Dalton Trans..

[59] Abrams M.J., Giandomenico C.M., Murrer B.A., Vollano J.F. (1991). Pt(IV) Complexes as Anti-Tumor Agents. U.S. Patent.

[60] Giandomenico C.M., Abrams M.J., Murrer B.A., Vollano J.F., Rheinheimer M.I., Wyer S.B., Bossard G.E., Higgins J.D. (1995). Carboxylation of kinetically inert platinum(iv) hydroxy complexes. an entree into orally active platinum(iv) antitumor agents. Inorg. Chem..

[61] Ojha R., Boas J.F., Deacon G.B., Junk P.C., Bond A.M. (2016). EPR spectroscopic characterization of a monomeric Pt^III^ species produced via electrochemical oxidation of the anticancer compound *trans*-[Pt^II^{(*p*-HC_6_F_4_)NCH_2_CH_2_NEt_2_}Cl(py)]. J. Inorg. Biochem..

[62] Ojha R., Nafady A., Shiddiky M.J.A., Mason D., Boas J.F., Torriero A.A.J., Bond A.M., Deacon G.B., Junk P.C. (2015). Conditions Favoring the Formation of Monomeric Pt^III^ Derivatives in the Electrochemical Oxidation of *trans*-[Pt^II^{(*p*-BrC_6_F_4_)NCH_2_CH_2_NEt_2_}Cl(py)]. ChemElectroChem.

[63] Kauffman G.B., Cowan D.O., Slusarczuk G., Kirschner S. (1963). cis- and trans-Dichlorodiammineplatinum(II). Inorganic Syntheses.

[64] Wilson J.J., Lippard S.J. (2014). Synthetic methods for the preparation of platinum anticancer complexes. Chem. Rev..

[65] Cox L.E., Peters D.G., Wehry E.L. (1972). Photoaquation of hexachloroplatinate (IV). J. Inorg. Nucl. Chem..

[66] Dunham S.O., Larsen R.D., Abbott E.H. (1993). Nuclear Magnetic Resonance investigation of the hydrogen peroxide oxidation of Platinum(II) complexes. crystal and molecular structures of sodium írans-dihydroxobis(malonato)plátinate(iv) hexahydrate and sodium frans-dihydroxobis(oxalato)platinate(iv) hexahydrate. Inorg. Chem..

[67] Murray P., Koch K.R., van Eldik R. (2014). Mechanism of tetrachloroplatinate(ii) oxidation by hydrogen peroxide in hydrochloric acid solution. Dalton Trans..

[68] Greenwood N.N., Earnshaw A. (1997). Chemistry of the Elements.

[69] Deacon G.B., Patrick J.M., Skelton B.W., Thomas N.C., White A.H. (1984). Ruthenium carbonyl complexes. III. Peparations, properties and structures of Dicarbonyl- and Monocarbonyl-(2,2′:6′,2″-terpyridyl)ruthenium(II) complexes. Aust. J. Chem..

[70] Ojha R., Junk P.C., Deacon G.B., Bond A.M. (2018). A supramolecular approach to the examination of the structures of some anticancer organoamidoplatinum(II) complexes. Supramol. Chem..

[71] Nakamoto K. (1986). Infra-Red and Raman Spectra of Inorganic Coordination Compounds.

[72] McPhillips T.M., McPhillips S.E., Chiu H.J., Cohen A.E., Deacon A.M., Ellis P.J., Garman E., Gonzalez A., Sauter N.K., Phizackerley R.P. (2002). Blu-Ice and the Distributed Control System: Software for data acquisition and instrument control at macromolecular crystallography beamlines. J. Synch. Rad..

[73] Kabsch W. (1993). Automatic processing of rotation diffraction data from crystals of initially unknown symmetry and cell constants. J. Appl. Crystallogr..

[74] Sheldrick G.M. (2008). A short history of SHELX. Acta Crystallogr. Sect. A.

[75] (2005). APEX2 v 2.0.

[76] Barbour L.J. (2001). X-Seed—A software tool for supramolecular crystallography. J. Supramol. Chem..

[77] Broughton D.B., Wentworth R.L., Laing M.E. (1947). Mechanism of decomposition of hydrogen peroxide solutions with manganese dioxide II. J. Am. Chem. Soc..

[78] Kukushkin V.Y., Belskii V., Aleksandrova E., Pankova E., Konovalov V., Yakovlev V., Moiseev A. (1991). Unusual Redox-Pairing of Acetoxime Ligands in Platinum Complex. Zhurnal Obs. Khimii.

[79] Melanson R., Rochon F.D. (1976). The crystal structure of potassium trichloro(2,6-lutidine)platinum(II). Can. J. Chem..

[80] Cingi Biagini M., Ferrari M., Lanfranchi M., Marchiò L., Angela Pellinghelli M. (1999). Chirality in mononuclear square planar complexes. J. Chem. Soc. Dalton Trans..

[81] Flack H.D. (1983). On Enantiomorph-Polarity Estimation. Acta Cryst..

[82] Cowieson N.P., Aragao D., Clift M., Ericsson D.J., Gee C., Harrop S.J., Mudie N., Panjikar S., Price J.R., Riboldi-Tunnicliffe A. (2015). MX1: A bending-magnet crystallography beamline serving both chemical and macromolecular crystallography communities at the Australian Synchrotron. J. Synchrotron. Radiat..

