# Supplementary material for: Oxidation of the Platinum(II) Anticancer Agent [Pt{(p-BrC6F4)NCH2CH2NEt2}Cl(py)] to Platinum(IV) Complexes by Hydrogen Peroxide
Source: Molecules. 2023 Sep 1;28(17):6402. doi: 10.3390/molecules28176402 (PMC10490441; doi:10.3390/molecules28176402)
Supplement: Supplementary file 1 [file molecules-28-06402-s001.zip › molecules-2563404-supplementary.pdf]

Supporting information

Oxidation of the platinum(II) anticancer agent [Pt{(*p*-$BrC_6F_4)NCH_2CH_2NEt_2$}Cl(py)] to Platinum(IV) complexes by hydrogen peroxide

Ruchika Ojha[1], Peter C. Junk[2]*, Alan M. Bond[1], Glen B. Deacon[1]

[1]School of Chemistry, Monash University, Clayton 3800 VIC, Australia, [2]College of Science, Technology & Engineering, James Cook University, Townsville, Qld, 4811, Australia.

E-mail: Alan Bond alan.bond@monash.edu, Glen Deacon glen.deacon@monash.edu, Peter Junk peter.junk@jcu.edu.au

Table of contents:

S1. Pt^{IV} data

Table S1 Selected bond angles for compounds **2·$H_2O_{(gold)}$** and **3·CH_2Cl_2**.

Figure S1 Comparison of Powder XRD patterns calculated from single crystal X-ray diffraction data of **2·$H_2O_{(gold)}$** and **2·$H_2O_{(red)}$** presented in the respective colors.

Table S2 Selected bond angles for compound **2·$H_2O_{(red)}$**.

S2. [NBu_4][$PtCl_3$(py)] Data

Figure S2 Molecular structure of [NBu_4][$PtCl_3$(py)], showing 50% thermal ellipsoids.

Table S3 Crystallographic data for the molecular structures of [NBu_4][$PtCl_3$(py)].

Table S4 Selected bond lengths and bond angles of [NBu_4][$PtCl_3$(py)].

S3. Variable temperature NMR spectra

Figure S3 ^{19}F NMR spectra obtained for **2·$H_2O_{(red)}$** over the temperature range of 25 °C to −60 °C.

Figure S4 ^{1}H NMR spectra obtained for **2·$H_2O_{(red)}$** over temperature range of 25 °C to −60 °C, showing 6-9 ppm region.

Figure S5 ^{1}H NMR spectra obtained for **2·$H_2O_{(red)}$** over the temperature range of 25 °C to −60 °C, showing 1 - 4.5 ppm region.

Figure S6 ^{19}F NMR spectra obtained for **2·$H_2O_{(red)}$** at 25°C and 50°C.

S4. PXRD data

Figure S7 Normalized powder X-ray diffraction data for bulk samples of **2·$H_2O_{(gold)}$** and **2·$H_2O_{(red)}$**, shown in **their** respective colors.

Figure S8 Normalized powder X-ray diffraction data for bulk sample of **2·$H_2O_{(red)}$** with normalized powder X-ray diffraction data generated from the single crystal of **1H** and **2·$H_2O_{(red)}$**.

Figure S9 Normalized powder X-ray diffraction data for bulk sample of **2·$H_2O_{(gold)}$** with normalized powder X-ray diffraction data generated from the single crystals of **1H** and **2·$H_2O_{(gold)}$**.

S5. Isolation of Pt^{IV} from the solution of an aged bulk sample

Table S5 Unit cell parameters for **1H** and co-crystallized **1(H/Cl)** collected from the bulk sample of **2·$H_2O_{(red)}$** and **2·$H_2O_{(gold)}$**.

Table S6 Unit cell parameters for Pt^{IV} **2·$H_2O_{(red)'}$**, co-crystallized **1(H/Cl)** and [NBu_4][$PtCl_3$(py)] isolated from the solution of an aged sample of **3·0.5CH_2Cl_2**.

S6. Electrospray MS measurements

S7. IR Spectroscopy data

S1. Pt^{IV} data

Table S1 Selected bond angles for compounds **2·$H_2O_{(red)}$** and **3·0.5CH_2Cl_2**.

**Bond angle**	**2·$H_2O_{(red)}$** **$C_{17}H_{23}BrClF_4N_3O_3Pt$** **(°)**	**3·0.5CH_2Cl_2** **$C_{17}H_{22}BrClF_4N_3O_2Pt$** **(°)**
**O1-Pt-O2**	177.7(3)	176.57(13)
**O1-Pt-$N1_{(amide)}$**	92.6(3)	90.96(13)
**O2-Pt-$N1_{(amide)}$**	85.3(4)	86.01(13)
**O1-Pt-$N2_{(amine)}$**	91.4(3)	93.11(13)
**O2-Pt-$N2_{(amine)}$**	89.1(3)	88.16(14)
**O1-Pt-$N3_{(py)}$**	88.0(3)	89.24(13)
**O2-Pt-$N3_{(py)}$**	91.4(3)	89.44(13)
**Cl-Pt-$N1_{(amide)}$**	176.4(3)	178.33(10)
**$N1_{(amide)}$-Pt-$N2_{(amine)}$**	83.9(3)	83.85(14)
**$N1_{(amide)}$- Pt-$N3_{(py)}$**	93.6(3)	95.15(13)
**Pt-$N1_{(amide)}$-$C7_{(deen)}$**	108.8(7)	108.1(2)
**$C7_{(deen)}$-$N1_{(amide)}$-$C6_{(C6F4)}$**	117.4(8)	117.9(3)
**Pt-$N2_{(amine)}$-$C8_{(deen)}$**	103.1(5)	103.3(2)

**Cl-Pt-N2$_{(Et2)}$**	94.9(2)	95.88(10)
**Cl-Pt-N3$_{(py)}$**	87.6(3)	85.05(10)
**N2$_{(Et2)}$-Pt-N3$_{(py)}$**	177.4(3)	177.45(13)
**Pt-N1$_{(amide)}$-C6$_{(C6F4)}$**	118.2(7)	116.9(3)

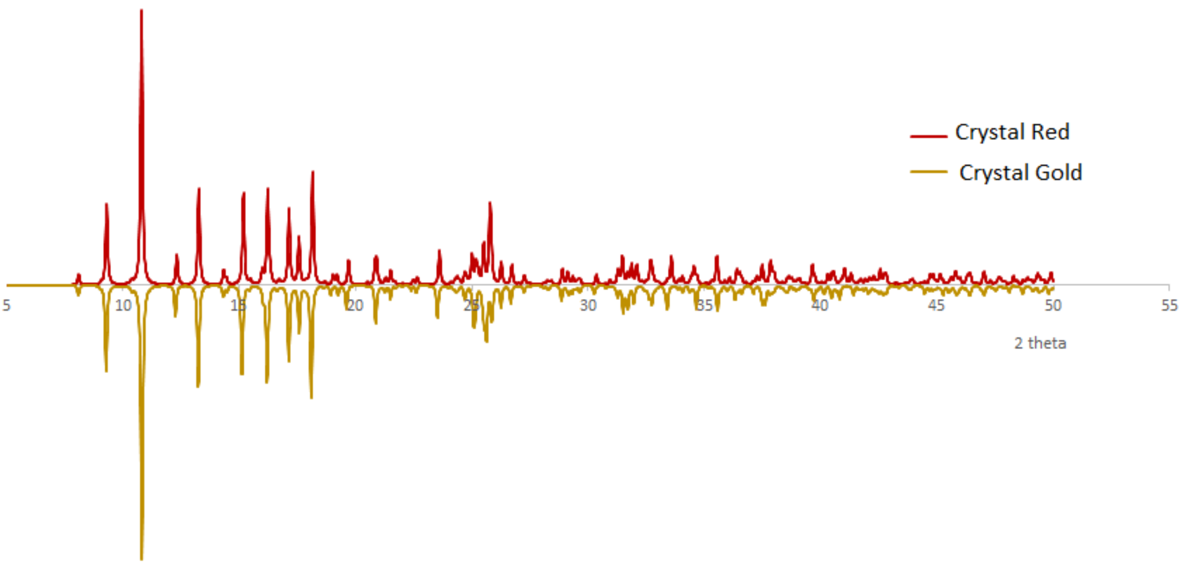

Figure S1 Comparison of Powder XRD patterns calculated from single crystal X-ray diffraction data of 2·$H_2O_{(gold)}$ and 2·$H_2O_{(red)}$ presented in the respective colors.

Table S2 Selected bond angles for compound 2·$H_2O_{(red)}$).

**Bond angle**	**2·$H_2O_{(red)}$ (°)<br>$C_{17}H_{23}BrClF_4N_3O_3Pt$<br>(molecules A and B in asymmetric unit)**	
**O1-Pt-O2**	177.9(3)	177.8(4)
**O1-Pt-$N1_{(amide)}$**	92.2(3)	91.9(4)
**O2-Pt-$N1_{(amide)}$**	85.7(3)	86.0(4)
**O1-Pt-$N2_{(amine)}$**	91.3(3)	90.8(3)
**O2-Pt-$N2_{(amine)}$**	88.8(3)	89.5(3)
**O1-Pt-$N3_{(py)}$**	91.9(3)	91.0(3)
**O2-Pt-$N3_{(py)}$**	87.9(3)	88.5(3)
**O1-Pt-Cl**	90.8(2)	90.8(2)
**O2-Pt-Cl**	91.2(2)	91.3(2)
**Cl-Pt-$N1_{(amide)}$**	176.5(3)	177.1(3)
**$N1_{(amide)}$-Pt-$N2_{(amine)}$**	84.0(4)	83.7(4)
**$N1_{(amide)}$- Pt-$N3_{(py)}$**	93.1(4)	93.2(4)
**Pt-$N1_{(amide)}$-$C7_{(deen)}$**	108.5(7)	108.6(7)
**$C7_{(deen)}$-$N1_{(amide)}$-$C6_{(C6F4)}$**	117.9(9)	119.0(9)

**Pt-N2$_{(amine)}$-C8$_{(deen)}$**	103.3(6)	103.9(6)
**Cl-Pt-N2$_{(Et2)}$**	95.1(3)	95.1(3)
**Cl-Pt-N3$_{(py)}$**	87.8(3)	88.0(3)
**N2$_{(Et2)}$-Pt-N3$_{(py)}$**	177.0(3)	176.8(4)
**Pt-N1$_{(amide)}$-C6$_{(C6F4)}$**	119.0(7)	118.4(7)
**N1-C7$_{(deen)}$-C8$_{(deen)}$**	105.4(9)	106.2(9)

S2. [NBu$_4$][PtCl$_3$(py)] Data

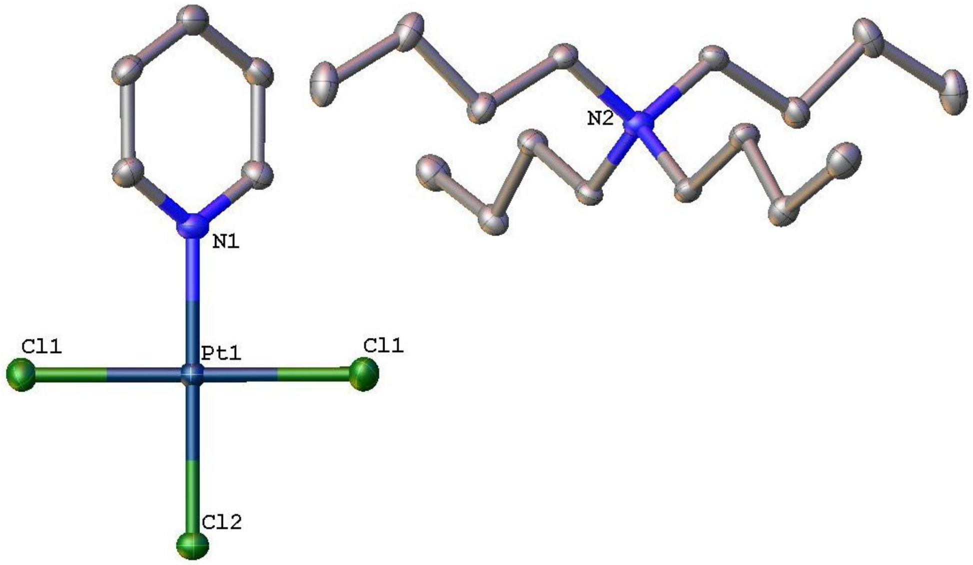

Figure S2 Molecular structure of [NBu$_4$][PtCl$_3$(py)], showing 50% thermal ellipsoids. H atoms are removed for clarity.

Similar structure to [NBu$_4$][PtCl$_3$(C$_5$H$_5$N)] are reported earlier such as [NEt$_4$][PtCl$_3$(C$_6$H$_5$N)] [S1] [K][PtCl$_3$(C$_6$H$_3$(CH$_3$)$_2$N)] [S2], [C$_5$H$_4$(C$_2$H$_5$)NH][PtCl$_3$(C$_6$H$_4$(C$_2$H$_5$)N)] [S3] etc. The bond lengths and angles of the anion [PtCl$_3$(C$_6$H$_5$N)]$^-$ are similar in all of these compounds.

Table S3 Crystallographic data for the molecular structures of [NBu_4][$PtCl_3$(py)].

	[NBu_4][$PtCl_3$(py)]
**empirical formula**	$C_{21}H_{41}Cl_3N_2Pt$
**Formula weight**	623.00
**Crystal system**	tetragonal
**Space group**	$P4_32_12$
a **(Å)**	14.337(2)
b **(Å)**	14.337(2)
c **(Å)**	12.083(2)
α **(°)**	90
β **(°)**	90
γ **(°)**	90
**vol(Å^3)**	2483.7(9)
**Z**	4
ρ **(calcd) (g/cm^3)**	1.666
μ **(mm^{-1})**	5.982
**F(000)**	1240
**reflections collected/ unique**	45812/3812
R_{int}	0.0663
$2\theta_{max}$ **(°)**	63.5
**Goodness-of-fit on F^2**	1.085
***R1* indices [I>=2σ (I)]**	0.0277
***wR2* indices [I>=2σ (I)]**	0.0694
**Absolute structure parameter**	0.025(3) [S4]

Table S4 Selected bond lengths and bond angles of [NBu_4][$PtCl_3$(py)].

Bond	length (Å)	Bond	Angle (°)
**Pt-Cl1**	2.3174(12)	N1-Pt-Cl1	89.91(3)
**Pt-Cl1′**	2.3174(12)	N1-Pt-Cl2	180.000(12)
**Pt-Cl2**	2.3078(15)	Cl1-Pt-Cl1′	179.81(7)
**Pt-N1**	2.003(5)	N1-Pt-Cl1	90.09(3)

S3. Variable temperature NMR spectra

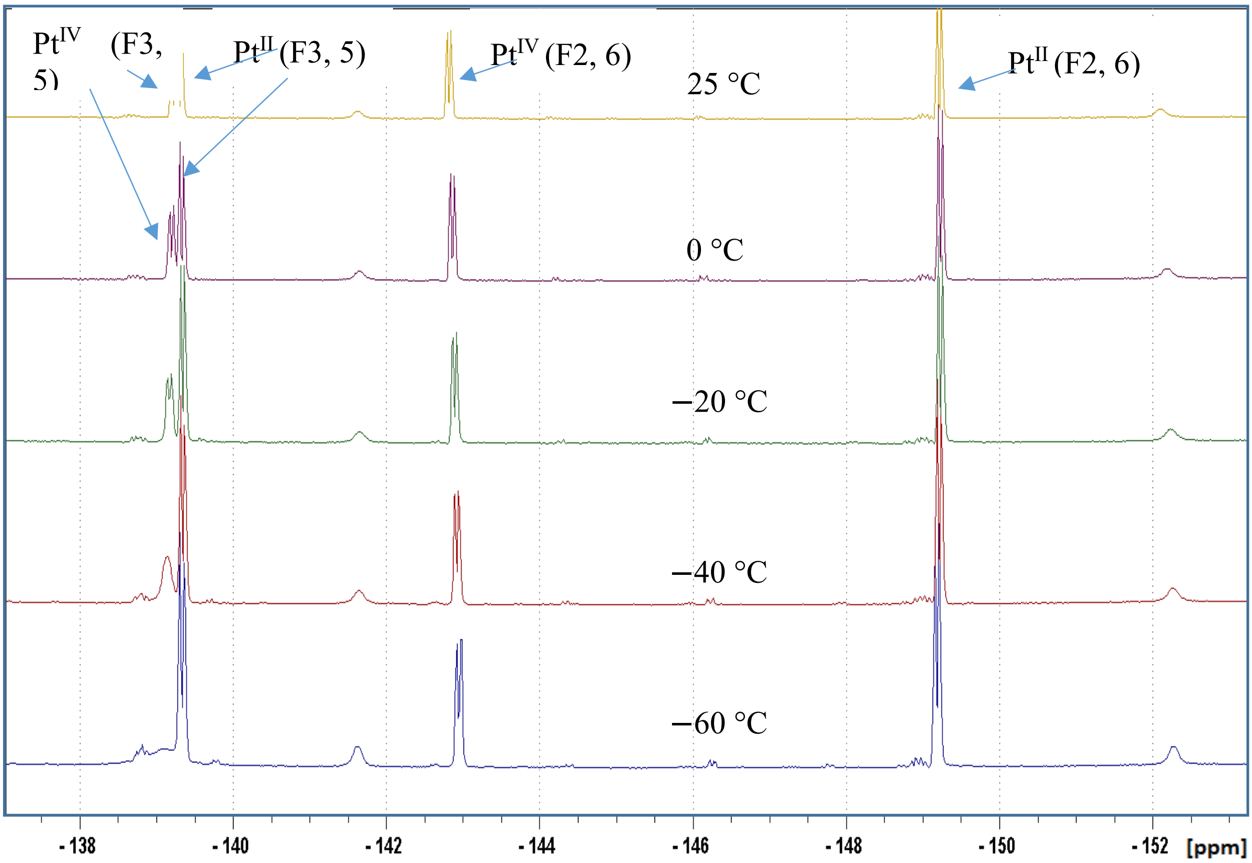

Figure S3 ^{19}F NMR spectra obtained for 2·$H_2O_{(red)}$ over the temperature range of 25 °C to -60 °C.

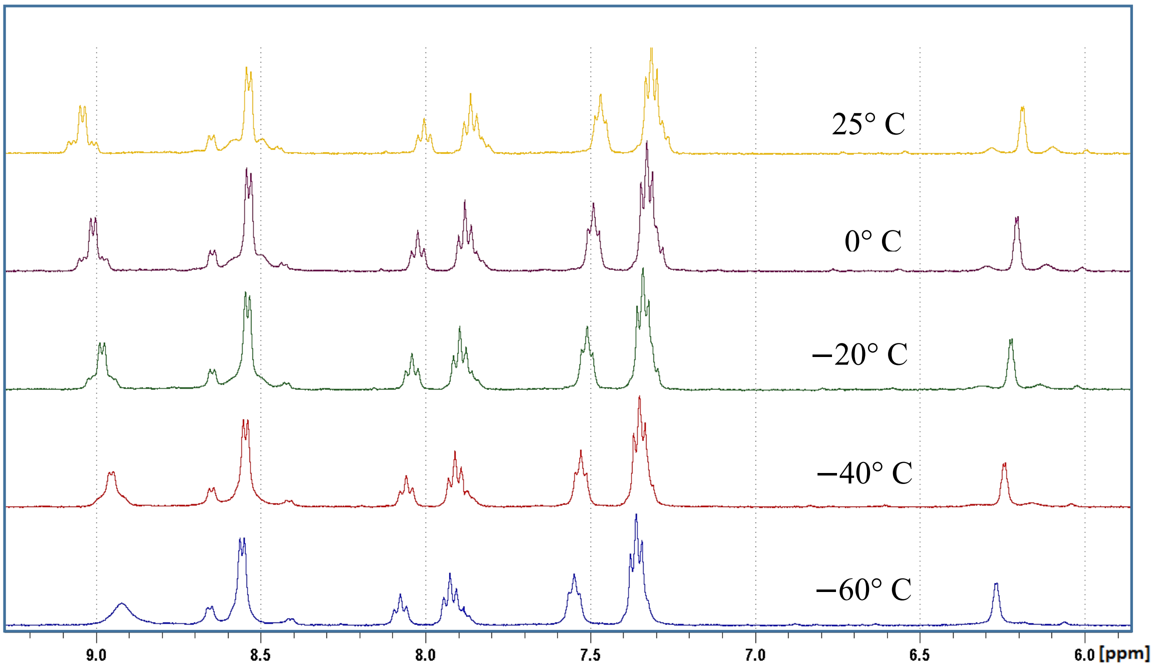

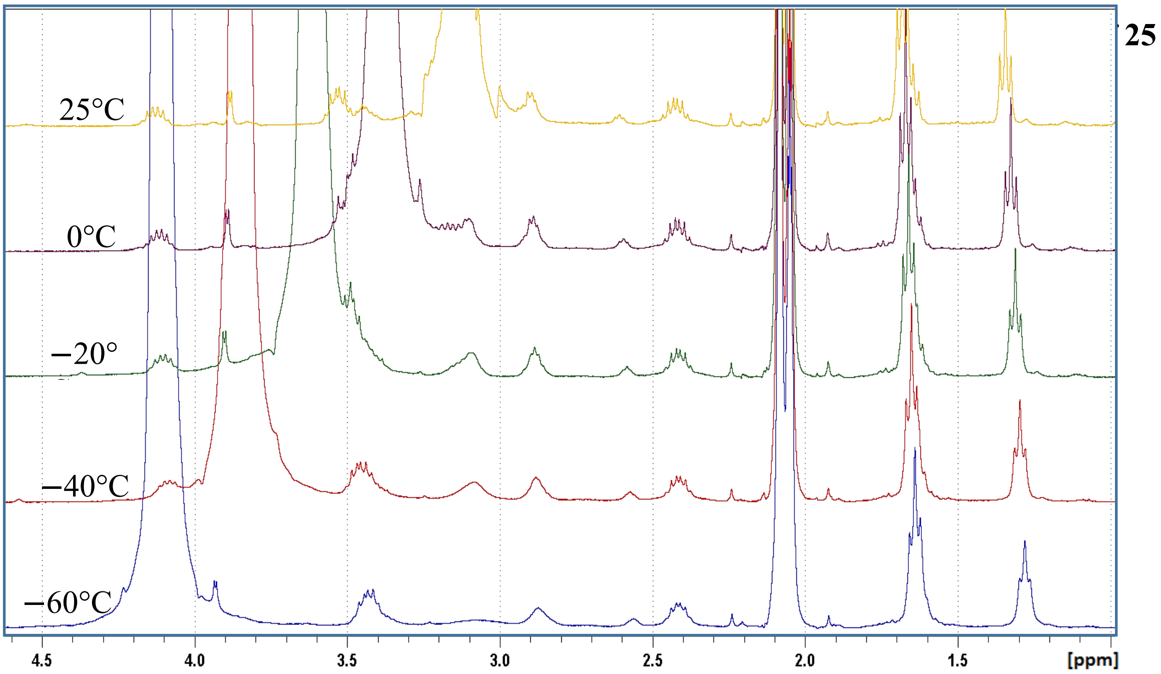

Figure S5 **^{1}H NMR spectra obtained for $2{\cdot}H_2O_{(red)}$ over the temperature rangeof 25 °C to −60 °C, showing 1 - 4.5 ppm region.**

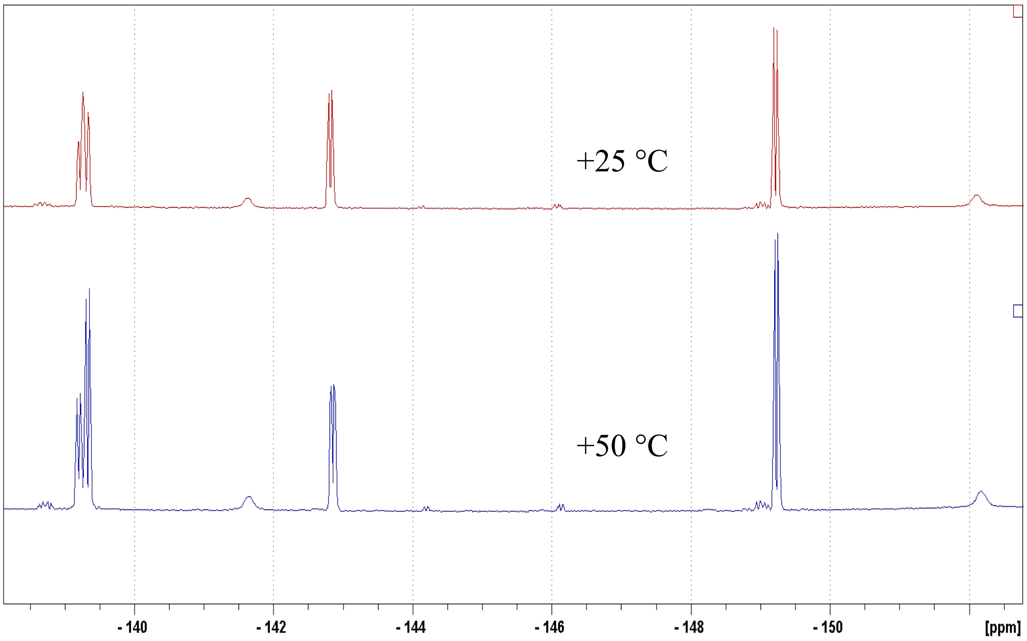

Figure S6 **^{19}F NMR spectra obtained for $2{\cdot}H_2O_{(red)}$ at 25°C and 50°C.**

S4. PXRD data

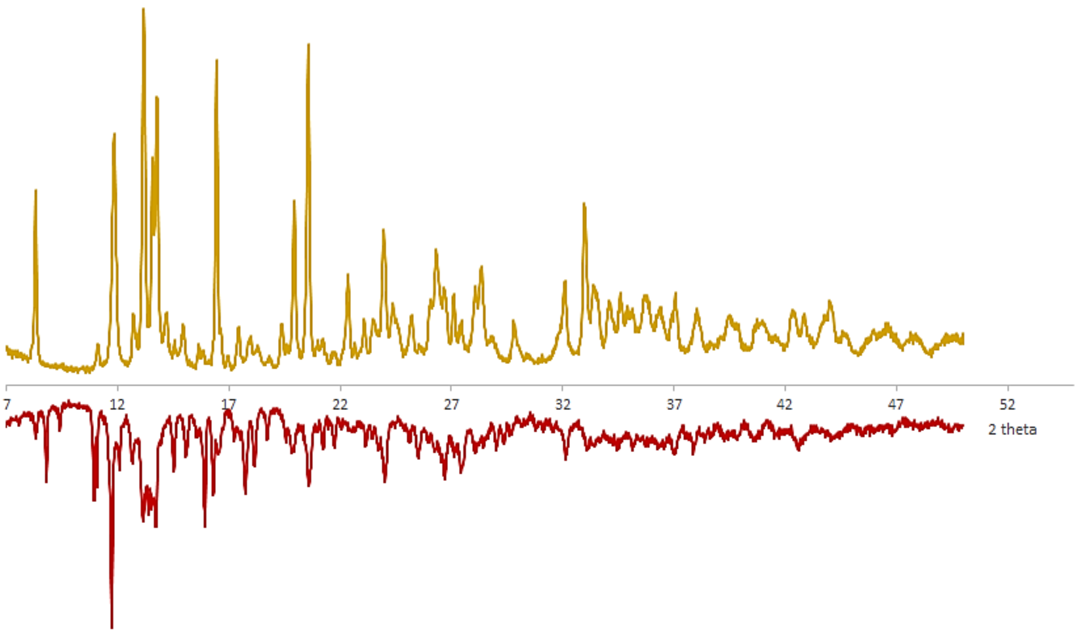

Figure S7 Normalized powder X-ray diffraction data for bulk samples of 2·$H_2O_{(red)}$ and 2·$H_2O_{(gold)}$, shown in the respective colors.

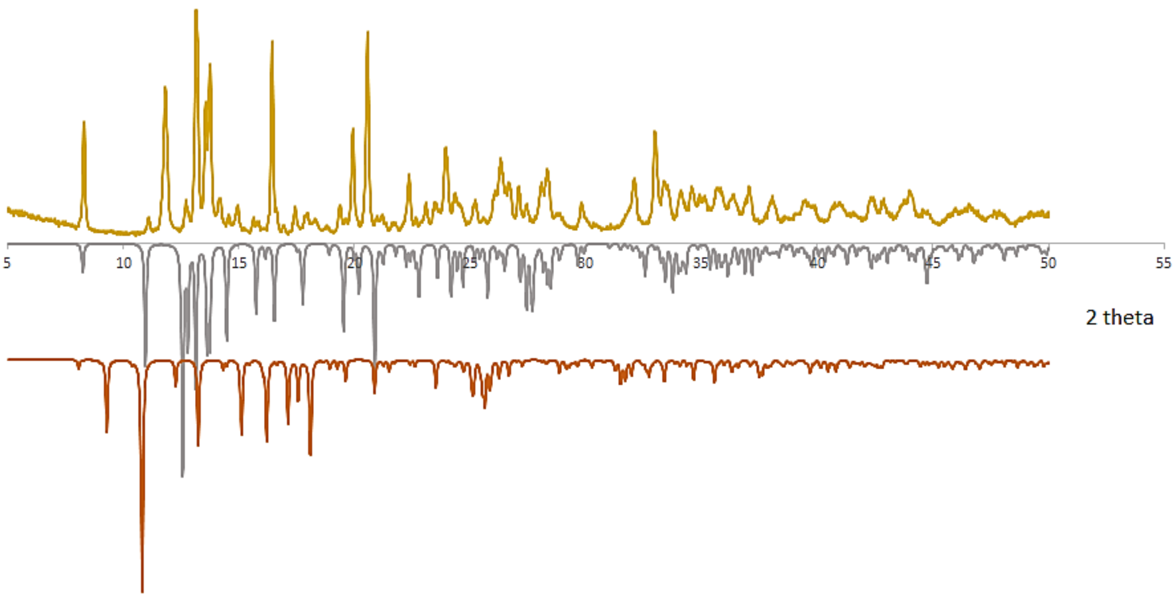

Figure S8 Normalized powder X-ray diffraction data for bulk sample of 2·$H_2O_{(red)}$ (in red), with normalized powder X-ray diffraction data generated from the single crystals of 1H (in grey) and 2·$H_2O_{(red)}$ (in orange).

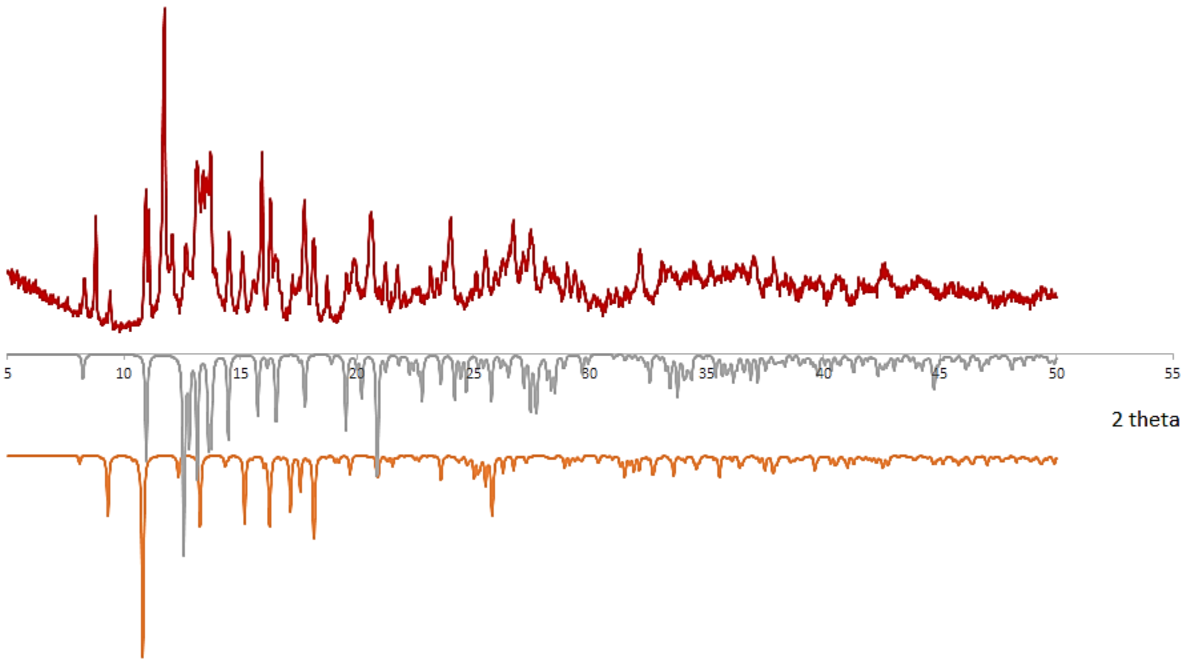

Figure S9 Normalized powder X-ray diffraction data for bulk sample of 2·$H_2O_{(gold)}$ (in gold), with normalized powder X-ray diffraction data generated from the single crystals of 1H (in grey) and 2·$H_2O_{(gold)}$ (in orange).

S5. Isolation of PtIV from the solution of an aged bulk sampleTable S5 Unit cell parameters for **1H** and co-crystallized **1(H/Cl)** collected from the bulk sample of **2·$H_2O_{(red)}$** and **2·$H_2O_{(gold)}$**.

	**2·$H_2O_{(red)}$′**	**Co-crystallized 1(H/Cl)**	**[NBu_4][$PtCl_3$(py)]**
a **(Å)**	19.6389(16)	8.5696(7)	14.3299(8)
b **(Å)**	13.3360(11)	21.8047(21)	14.3299(8)
c **(Å)**	16.9327(13)	10.4957(10)	12.0653(10)
α **(°)**	90	90	90
β **(°)**	104.857(2)	92.456(8)	90
γ **(°)**	90	90	90
**vol (Å^3)**	4286.5(6)	1959.4(3)	2477.6(3)

Table S6 Unit cell parameters for Pt^{IV} **2·H$_2$O$_{(red)}$′**, co-crystallized **1(H/Cl)** and [NBu$_4$][PtCl$_3$(py)] isolated from the solution of an aged sample of **3·0.5CH$_2$Cl$_2$**.

	**1H from 2·H$_2$O$_{(red)}$**	**1H[S5]**	**co-crystallised 1(H/Cl) from 2·H$_2$O$_{(gold)}$**	**1(H/Cl)[S5]**
a **(Å)**	8.5673(6)	8.5390 (17)	8.5852(7)	8.5861 (3)
b **(Å)**	10.6126(8)	10.613 (2)	21.8326(21)	21.9443 (9)
c **(Å)**	10.8595(15)	10.846 (2)	10.4934(10)	10.5178 (4)
α **(°)**	82.054(9)°	82.03 (3)	90°	90°
β **(°)**	85.527(8)°	85.47 (3)	92.431(8)°	91.856 (2)
γ **(°)**	87.451(6) °	87.28 (3)	90°	90°
**vol(Å^3)**	974.3(2)	969.7 (3)	1965.7(3)	1980.6813)

S6. Electrospray MS measurements

Similar to what was observed for *trans*-organoenamineamidoplatinum(II) complexes [S5], the starting material **1** was observed in the low-resolution mass spectra. However, in the accurate mass spectrum, only the (**1H** + H)$^+$ ion was obtained. For **2·H$_2$O$_{(gold)}$** or **2.H$_2$O$_{(red)}$**, no Pt^{IV} complex was detected in the low-resolution positive electron spray mass spectrum, and in the accurate mass spectrum, (**1H** + H)$^+$ was obtained. These results confirm the reductive dehydration of Pt^{IV} complexes into **1H**.

1H: ESI *m/z* (+ve): 652.2 (20% (**1**+H)$^+$); acc. Mass MS/ESI calc. for ((**1H**) + H)$^+$ i.e., ($C_{17}H_{17}BrClF_4N_3Pt$ + H$^+$): 649.9902, found : 649.9930. All these data agree with the reported **1H** [S5].

2·H$_2$O$_{(gold)}$: ESI *m/z* (+ve): 652.2 (100% (**1**+H)$^+$); acc. Mass MS/ESI calc. for ((**1H**)+H)$^+$ i.e., ($C_{17}H_{17}BrClF_4N_3Pt$ + H$^+$): 649.9902, found : 649.9930.

2·H$_2$O$_{(red)}$: ESI *m/z* (+ve): 652.2 (100% (**1**+H)$^+$); acc. Mass MS/ESI calc. for ((**1H**)+H)$^+$ i.e., ($C_{17}H_{17}BrClF_4N_3Pt$ + H$^+$): 649.9902, found : 649.9930.

S7. IR Spectroscopy data

1H: 3058w, 2962w, 2926w, 2868w, 2165w, 2080w, 1660s, 1619s, 1468s, 1451w, 1372m, 1355m, 1286w, 1263w, 1224m, 1190s, 1160w, 1111m, 1142s, 1087w, 1026m, 998m, 972s, 956m, 846w, 818s, 762s, 741s, 694s, 639w, 607s cm^{-1}. All these data agree with the reported **1H** [S5].

References

S1. Kukushkin, V. Y.; Belskii, V.; Aleksandrova, E.; Pankova, E.; Konovalov, V.; Yakovlev, V.; Moiseev, A., *ZHURNAL OBSHCHEI KHIMII* **1991,** *61*, 318-328.

S2. Melanson, R.; Rochon, F. D., The crystal structure of potassium trichloro(2,6-lutidine)platinum(II). *Can. J. Chem.* **1976,** *54*, 1002-1006.

S3. Cingi Biagini, M.; Ferrari, M.; Lanfranchi, M.; Marchiò, L.; Angela Pellinghelli, M., Chirality in mononuclear square planar complexes. *J. Chem. Soc., Dalton Trans.* **1999,** 1575-1580.

S4. Flack, H. D., *Acta Cryst.* **1983,** *A39*, 876-881.

S5. Ojha, R.; Mason, D.; Forsyth, C. M.; Deacon, G. B.; Junk, P. C.; Bond, A. M., *J. Inorg. Biochem.* **2021,** *218*, 111360.
